# Cyanobacteria and Macroinvertebrate Relationships in Freshwater Benthic Communities beyond Cytotoxicity

**DOI:** 10.3390/toxins16040190

**Published:** 2024-04-15

**Authors:** Nicolás Ubero-Pascal, Marina Aboal

**Affiliations:** 1 Department of Zoology and Physical Anthropology, Faculty of Biology, Espinardo Campus, University of Murcia, E-30100 Murcia, Spain; nubero@um.es; 2 Laboratory of Algology, Faculty of Biology, Espinardo Campus, University of Murcia, E-30100 Murcia, Spain

**Keywords:** cyanobacteria, cyanotoxins, benthos, macroinvertebrates, detoxification, accumulation

## Abstract

Cyanobacteria are harmful algae that are monitored worldwide to prevent the effects of the toxins that they can produce. Most research efforts have focused on direct or indirect effects on human populations, with a view to gain easy accurate detection and quantification methods, mainly in planktic communities, but with increasing interest shown in benthos. However, cyanobacteria have played a fundamental role from the very beginning in both the development of our planet’s biodiversity and the construction of new habitats. These organisms have colonized almost every possible planktic or benthic environment on earth, including the most extreme ones, and display a vast number of adaptations. All this explains why they are the most important or the only phototrophs in some habitats. The negative effects of cyanotoxins on macroinvertebrates have been demonstrated, but usually under conditions that are far from natural, and on forms of exposure, toxin concentration, or composition. The cohabitation of cyanobacteria with most invertebrate groups is long-standing and has probably contributed to the development of detoxification means, which would explain the survival of some species inside cyanobacteria colonies. This review focuses on benthic cyanobacteria, their capacity to produce several types of toxins, and their relationships with benthic macroinvertebrates beyond toxicity.

## 1. Introduction

Most published works on the interrelationships between cyanobacteria and other freshwater organisms tend to demonstrate and quantify the harmful effect of cyanobacteria and how other freshwater organisms attempt to survive them [1,2]. However, the stress situations that cause massive cyanobacteria growth and the associated toxicity are usually extraordinary in ecosystems: floods, droughts, eutrophication, deforestation, water temperature changes, competition for nutrients, etc. [3]. Toxic events where cyanobacteria can be found or are involved are not new, but have occurred since ancient times. For example, molecular studies have revealed the presence of cyanotoxins in sediments dating back to the ancient Mayan culture (550–1200 years ago), and are associated with algal bloom episodes [4]. References to color changes to fresh water associated with fish mortality appear in the Book of Exodus and are collected in 12th-century manuscripts in Scotland, in which monks refer to these episodes as “sick lochs” [5] More recently, animal poisoning or deaths related to cyanobacteria toxicity have been noticed worldwide, with examples in Australia [6] and Poland [7]. In a climate or global change context, the stress situations that trigger the toxic response of cyanobacteria will probably become more frequent and harmful worldwide in the future [8] and will continue to be extraordinary. Despite their controversial reputation, cyanobacteria are among the first inhabitants on our planet, and have contributed to create favorable environmental conditions for life on Earth and its diversification [9], and have established numerous symbiotic relationships with other organisms, from unicellular eukaryotes to aquatic and terrestrial metaphytes and metazoans [10]. They also play a determinant role in the maintenance of freshwater ecosystems, especially lotic ones, but this aspect is still poorly understood [11].

Cyanobacteria can be collected and might live in a wide variety of habitats, including extreme conditions, because of their adaptations in nutrient storage, N fixation, buoyancy, formation of resting cells, UVR-protective compounds or the production of wide sheaths [9]. It is important to remember that blue–green algae may capture atmospheric nitrogen (N) in either specialized cells (heterocytes) or vegetative cells in special environmental conditions. Cyanobacteria’s capacity to produce cyanotoxins was acquired very early on. Molecular sediment studies evidence the presence of microcystins (MCs) and cylindrospermopsin (CYN) 4700 years ago [12] and that of saxitoxins (SXT) 2.1 billion years ago [13]. This ability to synthesize toxins was missed or retained by different taxa during the group’s phylogenetic history [14].

The keenest interest shown in cyanotoxin studies has always been related to planktonic species, such as animals (including humans) that depend on reservoirs and lakes for water supply, yet this fact might lead to some bias in the knowledge and interpretation of cyanotoxicity. For instance, besides believing that cyanotoxins are linked with stress conditions in high eutrophic masses of water, the presence of these compounds has also been reported in unpolluted calcareous rivers and oligotrophic reservoirs and lakes [15,16,17,18]. Although our understanding of benthic cyanobacterial species’ diversity and distribution in freshwater streams is improving worldwide, their ability to produce cyanotoxins has been poorly studied.

Among living organisms, cyanobacteria are one of those that produce a high diversity of toxins, which vary in terms of both their molecular nature and their main effects. Of cyanotoxins, MCs and nodularins (NODs) have been thoroughly studied, more than 246 isoforms of MCs have been identified [19], and both are cyclic peptides with hepatotoxic activity. However, cyanobacteria may also produce: alkaloids, such as CYN, with hepatotoxic, cytotoxic, dermatotoxic, and even possible carcinogenic properties [20]; anatoxins, mainly anatoxin-a (ATX-a), with neurotoxic capacity [21]; SXT, which are one of the most potent naturally-occurring neurotoxins, but have been associated only with marine environments and Dinophyta until quite recently [22]; and BMAA (β-N-methylamino-L-alanine), a neurotoxic nonprotein amino acid related to several neurodegenerative diseases [23].

Vertebrate exposure to toxins occurs mainly through drinking water or food consumption [20], with recreational water use as a secondary route in humans. The effects of cyanotoxins on other organisms have been reported, and are positive or negative depending on the species, taxonomic group, or environmental conditions [24,25]. Nonetheless, experimental design is sometimes not representative of what is expected to be found in nature in terms of toxin concentration or potential synergistic or antagonistic relationships if the production of several toxins happens at the same time [26]. As most data from toxicological studies refer to very high concentrations (most unlikely under natural conditions) and atypical exposure routes, future efforts should be made to observe environmentally relevant concentrations and oral and chronic exposures [26,27] to gain a clearer idea of the risks that biota and populations face.

The bioconcentration of toxins along food webs has long since been considered one of the major environmental problems. Apparently however, it does not always occur and biodilution might happen [28,29]. The intensity or frequency of both phenomena is poorly known. However, the heterogeneous bioaccumulation levels that benthic macroinvertebrates present, the different forms of exposure to cyanotoxins to which they are subjected, and the purification and detoxification processes that are beginning to be known will condition the transfer capacity of toxicity in the freshwater food chain, and even its export to the terrestrial food chain [30,31,32].

This review focuses on benthic cyanobacteria and their capacity to produce several types of toxins, the dependence of aquatic macroinvertebrates on benthic cyanobacteria, their biological relationships beyond toxicity, and future perspectives.

## 2. Results

### 2.1. Benthic Toxicity

The detection of MCs in benthic cyanobacteria was reported for alpine lakes in situations related to domestic animal deaths in 1997 [15]. At the beginning, most researchers thought that this was an exceptional case. However, when more people became interested in benthos, toxicity reports increased worldwide. Today, we know that benthic toxicity is widespread in all continents (geological units) and in all, or almost all, sorts of habitats [33], ranging from lagoons, rivers, springs, peat bogs and caves to a wide range of geographical and environmental conditions. This is consistent with the fact that cyanobacteria may colonize all types of substrata in all climatic and environmental conditions (except for low-pH waters), and toxicity is likely to be much more commonplace than previously thought. The concentration of toxins is always relatively low, but the presence of several variants is common in most producer genera.

**Figure 1:**
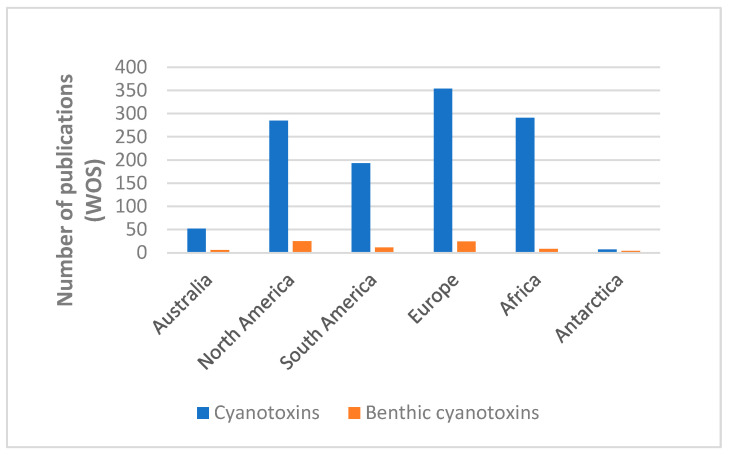
Distribution of papers published from 1997 on cyanotoxins and benthic cyanotoxins referring to continents or geological units.

Wood et al. [2] compared the number of publications on benthic and planktonic cyanotoxicity. Although references to benthic communities have considerably increased lately, there is still a huge difference between them. If we make a comparison of continents, the image is similar, with North America and Europe presenting higher number of publications on benthic cyanotoxins (Figure 1) but with numbers still very far from planktic studies.

The level and concentration of toxicity and toxins vary vastly in different countries. China and Canada host water bodies with the highest level of MCs [34], much higher than the World Health Organization’s permissible level of 1 μg/L. In fact, countries like Canada and Australia have raised the admissible MC concentration for drinking water to 1.5 and 1.3 μg/L, respectively [35,36], but the US National Center for Environmental Assessment claims that the WHO drinking-water guideline value should be lower [37].

Comparison of quantitative data from the literature poses a problem, because neither raw materials (from the field or grown in the laboratory) nor the identification and quantification methodology (biochemical or genomic) is similar. In any case, current evidence for the spread of benthic cyanotoxicity is overwhelming.

**Figure 2:**
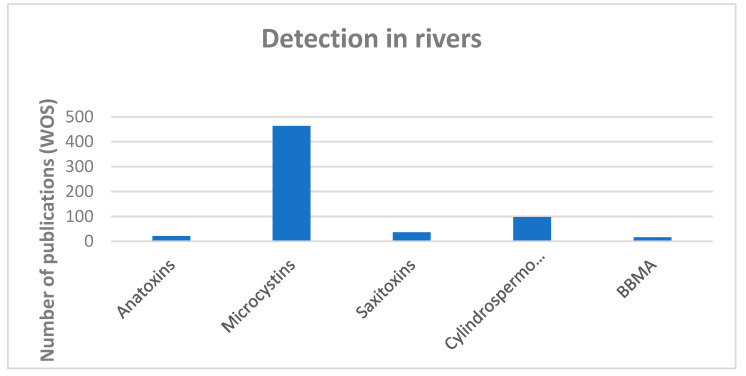
Papers published from 1997 related to the main groups of cyanotoxins and their detection in rivers.

Limnological or phycological studies have traditionally focused on lentic habitats, and lotic environments have usually been much less studied [2]. However, almost all possible cyanotoxins have been reported in rivers (Figure 2), and the number of studies that detect several toxic compounds in the same benthic samples is increasing [38,39,40,41,42,43]. It is not uncommon to find that several toxic congeners are present in the same localities and biofilms, and subdominant taxa sometimes produce higher concentrations of toxins [43,44,45,46]. This highlights the need to monitor not only biofilms where cyanobacteria are dominant.

Even when the parameters promoting toxin production are very likely the same, the factors involved in toxin release may differ vastly between lentic and lotic habitats, especially if the high diversity of aquatic system typologies is considered. Most lotic habitats are exposed periodically to drought and floods, and not only does their physiognomy change, but they have a marked effect on destruction and potential toxin release [9].

The detection of MCs in alpine lakes [15] was probably the first reference to MCs under oligotrophic conditions. The presence of toxins in oligotrophic calcareous rivers was reported later, and a correlation was then found between cyanobacteria biofilm toxicity and macroinvertebrate diversity by the *Photobacterium phosphoreum* test [47]. Several MC variants were identified in the same rivers, and significant negative correlations were found between the total intracellular MC content and air temperature, flow, and depth, while dissolved MCs increased with low depth and high flows but showed no significant correlation [17]. This scenario suggests a potential relationship with several environmental and climate-related variables rather than eutrophication, as confirmed later by paleolimnological data [48,49,50].

Heterocystous cyanobacteria (Nostocales) may fix N from the atmosphere and become independent of the N concentration of water [51], but Oscillatoriales and Chroococales can also do this under conditions with a low oxygen concentration [52,53]. However, the responses of fixing and non-fixing organisms to N/P ratios are sometimes paradoxical [54]. As the latter authors stated, the main problem is probably expecting homogeneous behavior in such a diverse group of organisms.

### 2.2. Multitoxic Biofilms

Most research efforts have focused on the effect of selected toxins, and not on what is probably the commonest case in nature: the presence of several toxins at the same time [38]. The potential synergy among cyanotoxins or the presence of other unknown toxic compounds cannot be ruled out, because the toxic effects of extracts are always stronger than pure toxic compounds [44,45].

Cyanobacteria blooms and biofilms are usually formed by several different strains, with potentially distinct requirements, and are both toxic and nontoxic, although the toxic ones are usually less frequent [55]. Detecting several toxic compounds in the same benthic community is also becoming increasingly common: Bouma-Gregson et al. [40,41] quantified MCs and anatoxin-a (ATX) in the Eel River (Angelo Coast Range Reserve, CA, USA). Carpenter [42] identified and quantified SXT, MCs, ATX and CYL in different taxa from the Clackamas Basin in Oregon (USA). Fadness et al. [38] quantified ATX, CYL, MC, NOD and SXT in benthic cyanobacteria in several Northern California rivers (samples from 2016–2019). The neurotoxins anatoxin-a (ATX), SXT and BMAA have also been reported from freshwater cyanobacteria [22,56,57,58]. No clear relationship to nutrients and cyanotoxicity has been found, as some other authors suggest [59].

Saxitoxin has been reported previously only from marine habitats, and anatoxin and BBMA are only known to be produced by freshwater (or soil) cyanobacteria [58,60]. However, we are now aware that saxitoxin and anatoxin have a widespread distribution, with reports on every continent, except Antarctica [22], but their distribution will very likely grow when more research has been conducted. BMAA seems to be present in all the morphological cyanobacteria groups from freshwater, brackish and marine environments [58]. As far as we know, there is no information about the effects of these toxins on river biota, but BMAA is related to several neurodegenerative diseases and STX has not been related to any human intoxication to date [1,58].

### 2.3. The Role of Mucilage

Mucilage production is important in the formation of cyanobacteria biofilms and colonies attached to rocks in river riffles. Mucilage might also play a role in the retention of nutrients and water (upon emersion), but it would seem that it can also retain toxins, as shown by Young et al. [61] and Marco et al. [62], who followed immunological methods. The retention of toxins by mucilage also seems to be common in other toxic groups, such as Dinophyta (very common in marine habitats) [63] and might represent a defense mechanism.

The relationship of mucilage and phosphorus (P) deficiency and the activity of phosphatases (mono- or diesterases) have been verified in several algal and plant groups [64,65]. It is important for the survival of all microalgae, including cyanobacteria, in calcareous habitats where P is retained in carbonate deposition.

### 2.4. Toxicity and Taxonomy

The generalization of applying the analysis of sequences to ensure the identity of organisms has revolutionized the taxonomy of all groups, including cyanobacteria, where the scarcity of diagnostic characteristics has always been a big problem and a challenge for taxonomists.

The implementation of a multiphasic approach with taxonomic, biochemical, ecological, and genomic information has been proposed in an attempt to gain a more complete image of taxa and their requirements [66], but this path has not been followed by all scientists. In the last few years, the genus *Nostoc* has been split into 15 genera: *Aliinostoc*, *Amazonocrinis*, *Atlanticotrix*, *Compactonostoc*, *Dendronalium*, *Desikacharya*, *Desmonostoc*, *Halotia*, *Komarekiella*, *Mojavia*, *Minunostoc*, *Parakomarekiella*, *Pseudoaliinostic*, *Purpureonostoc* and *Violetonostoc*. In addition, multiple new species have been described, with more than 100 recognized [67,68], which confirms much higher diversity than previously thought. Thus, caution is recommended when interpreting the literature to take into account nomenclatural changes. Without a clear morphological description or images, and no genomic information available, it is difficult to be sure of the names indicated in papers, and it is even more difficult to accurately make comparisons of toxicological aspects.

Can we now be sure that similar morphotypes belong to the same species and have a similar chemical composition and the ability to produce, or not, toxins?

Conspicuous *Nostoc pruniforme* colonies play an important role in the physiognomy and development of benthic communities in some lakes or rivers, and they may produce toxins. Recently, Carpenter [42] reported the presence of several different types of toxins in this species. In some Greenland lakes, *N. pruniforme* develops very big monospecific communities that produce toxins, which are released in different ways: grazing, active release, high nutrient concentration or physical disturbance [69].

There is a clear parallelism with calcareous streams, where floods that can seasonally occur destroy colonies and mats by releasing intracellular toxins [70].

### 2.5. Relationships to Benthic Macroinvertebrates

**Figure 3:**
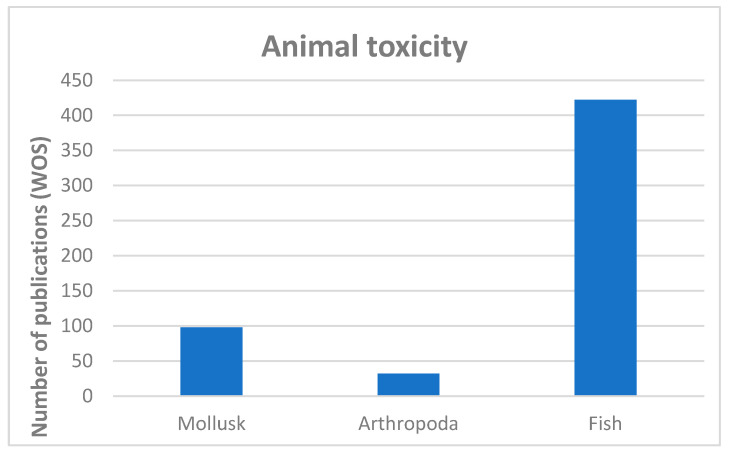
Papers published from 1997 related to the effect of cyanotoxins on different group of animals.

The biological relationship between animals and cyanobacteria in freshwater ecosystems remains intricate despite the numerous published studies that share both terms, or their derivatives, as keywords (Figure 3). Most of these papers tend to study the noxious effect (mainly lab-induced) of cyanobacteria on animals, their bioaccumulation or their possible transfer through the food chain [1,2,71]. Vertebrates, including humans, are the main group in which cyanotoxicity has been studied. If we focus on aquatic species, references accumulate from fish, while information about freshwater invertebrates is scarce (Figure 3), especially about benthic fauna [3,72]. However, very little is known about the role that benthic cyanobacteria–animal relationships play in the proper functioning of freshwater ecosystems, even though they have normally cohabited in these habitats for a long time [2]. Indeed, the role played by cyanobacteria in freshwater ecosystems must definitely be more important and complex than their simple capacity to produce toxins and to be harmful for other organisms [1].

Dudley et al. [73] proposed three possible pathways of ecology interrelationships among macroalgae, including cyanobacteria colonies, and macroinvertebrates in benthic stream ecosystems: (a) food source; (b) altering the habitat’s physical conditions, and even generating new ecological niches; and (c) competing for space. Food sources seem to be the principal interaction between cyanobacteria and invertebrates, and defense against grazing is one of the probable causes for which cyanobacteria may produce and secrete toxins to the environment [74,75]. Some authors consider this factor to be the least important [2]. Cyanobacteria are important primary producers from aquatic systems and can sometimes, in special environmental or seasonal circumstances, represent the main autotroph group and be the only food resource for freshwater invertebrates [76,77,78]. Calcareous oligotrophic rivers are colonized most of the year by a diverse cyanobacterial community, especially in Mediterranean areas [11,17]. Cyanobacteria have been traditionally considered a poor food resource, not only because they may produce toxins or present morphologies that are unappealing or difficult to ingest, such as long or thick filaments or mucilage, but also because they have been considered to be of low nutritional quality [74,79,80,81]. However, cyanobacteria contain nutrients and active macromolecules, such as pigments, carbohydrates, lipids (including essential fatty acids), proteins, vitamins, and minerals, which are necessary for the growth and maturation of macroinvertebrates [76,77,81,82,83]. A gut content analysis (microscopy, serology or DNA) has proven that certain grazer invertebrates feed on cyanobacteria [30,78,83,84,85,86,87,88,89,90,91,92], and they even prefer filamentous species [84,86,90,93], but their ingestion in other grazers may accidentally occur because some cyanobacteria species are usually found as epiphytes of macrophytes, or as part of complex biofilms, periphyton and detritus [2,3,83,94,95]. This dichotomy has led us to wonder whether the simple detection of cyanobacteria in grazers’ gut contents can be nutritionally considered by proposing an enzyme analysis as the most appropriate way to confirm that invertebrates have the capacity to digest cyanobacteria and to absorb their nutrients [84]. Cases of undigested cyanobacteria being eliminated in feces are described, and the culture of such debris may inform about the digestibility of ingested cyanobacteria [96,97]. Nevertheless, as certain cyanobacteria are able to fix atmospheric N, the nutritional value of cyanobacteria in some invertebrates, and also for the whole food web, has been revealed by studies of stable carbon (C) and N isotopes [81,95,98,99,100]. Studies of protein and lipids, especially fatty acid biomarkers, have also confirmed that cyanobacteria are essential for macroinvertebrate survival, especially in winter when no other food is within reach [70,95]. Benthic macroinvertebrates can eat cyanobacteria from benthos and plankton [101,102].

The trophic relationship between cyanobacteria and benthic invertebrates should not be limited only to grazers, but extended to detritivores. Deposits of decomposing organic matter in freshwater ecosystems may contain cyanobacteria in the form of living colonies and organic debris or by-products, such as their toxins [80,103,104]. Although some cyanotoxins released to the environment can be degraded through physical processes, such as photodegradation, other cyanotoxins might remain active for long periods of time when they reach sediment [1,105,106,107]. Furthermore, this detritus is the main way for benthic invertebrates to encounter planktonic cyanobacteria or their toxins, especially in lentic habitats (lakes, dams, reservoirs, etc.) or larger rivers after bloom episodes. Stepanian et al. [108] consider the presence of cyanotoxins in sediment to be one of the factors that could explain the decline of some benthic macroinvertebrates in lakes. Woller-Skar et al. [107] noted three factors that increase the likelihood of planktonic cyanobacteria in benthos: (a) incomplete spring recruitment; (b) falling out of suspension during the growing season; and (c) remaining viable after burial. Therefore, shredder and collector macroinvertebrates can also ingest cyanobacteria because they do not usually discriminate detritus components in food, which appear in gut contents as a minority component [84,91]. Some of them, however, show preferential food selection for cyanobacteria mats [86]. The microorganisms present in detritus, such as fungi and bacteria, play a fundamental role in not only the degradation and stabilization of organic matter, which is necessary for its assimilation by some detritivores, but also in the detoxification caused by the presence of cyanotoxins, especially MCs [109,110]. Several freshwater environmental microorganisms are capable of completely degrading MCs, and even of acting on their adda ring, and different enzymatic pathways have been found. Nonetheless, the most studied pathway is that by which microcytinase (MlrA) forms a part and involves a cluster of four genes (mlrABCD) [109,111,112].

The relationship between cyanobacteria and benthic macroinvertebrates is not only trophic, but they can also interact biologically by simply living in the same habitat. As is known, invertebrates can be affected by cyanotoxins through their ingestion, either diluted in water and associated with particles (sediment or cyanobacterial cells) or by contact and diffusion through integument, eggs or gill membranes [30,86,97,101,102,103,107]. The harmful effect of this cohabitation has been studied more in plankton than in benthic invertebrates [72,94]. The colonial forms of cyanobacteria or assemblages with other algae in complex mats can act as effective ecological niches, which can be exploited by macroinvertebrates. Some macroinvertebrates develop territorial behavior on cyanobacteria mats [79,113], while others prefer living among cyanobacteria colonies as a form of defense [72,113,114] or settle on top of them to be more exposed to currents or the water column [73]. In the most extreme case, a few macroinvertebrate species, such as chironomid *Cricotopus* spp., must live in *Nostoc* spp. colonies to survive by establishing a mutualistic relationship: the midge obtains shelter and an unlimited source of food, and cyanobacteria gain a fixation to substrate thanks to the silk secreted by larva, an increased photosynthesis surface and higher dispersive capacity [85,93,96,114,115,116,117]. Other dipteran species, like Ephydridae, can pierce cyanobacterial mats to live in them and condition the physiostratigraphy of soil on the shores of lakes, even on a geological scale [118]. Oncoids (calcareous stromatolites produced by cyanobacteria) and vertical rocky substrates (freshwater walls extensively colonized by cyanobacteria) constitute real small-scale ecosystems where a complex biocenosis, with a high diversity of invertebrates, establishes its own trophic relationships [114,119]. Moreover, algae composition or abundance in benthic habitats may condition macroinvertebrate biodiversity [94], and vice versa [79], but this ecological aspect has been poorly studied.

Benthic cyanobacteria produce all the types of cyanotoxins described in planktonic cyanobacteria, namely, hepatotoxins (MCs, NODs and CYN), neurotoxins (SXT, ATX-a and homoanatoxin-a) and dermatotoxins (lyngbyatoxin) [2,44,69,94], which cause different kinds of damage in benthic macroinvertebrates; e.g., molecular, cellular, tissue, metabolic, functional, developmental, etc. [2,32,105,120,121]. Interestingly, hepatopancreas seems to be the main target organ in Crustacea and Mollusca independently of cyanotoxins [2,30,99,101,122,123,124,125], but only occurs in muscle when toxin exposure thresholds are reached [30]. Curiously, some studies have highlighted that most cyanobacteria extracts, and even those species or strains that do not produce toxin, are more harmful than purified cyanotoxins by showing that cyanobacteria contain other toxic compounds beyond known toxins [2,31,90,123]. Toxicity in cyanobacteria is not taxa- but clone-related, and by considering the polyphyly in some genera like *Nostoc*, it should be elucidated if toxin producers and nonproducers belong to the same species [126]. Studies have already shown that the same species can produce toxins, or not, and even the same species may produce different toxins according to the geographical location or the physic-chemical parameter of habitats [2,80]. Although less studied than zooplankton, the harmful effects of cyanotoxins on benthic habitats depend on toxin type, invertebrate species or life cycle instars. However, the negligible presence or absence of mortality of some macroinvertebrates exposed to free cyanotoxins or cyanobacteria extracts, or which feed directly on cyanobacteria toxic strains, has suggested that they present a different degree of sensitivity, tolerance or resistance, and even certain species specificity [74,81,102]. Delaney and Wilkins [127] noted that the lethality of MCs for several land insects (larvae and adults), also observed in freshwater invertebrates, occurs in the long term after exposure or ingestion compared to mammals. This finding demonstrates the existence of differences in sensitivity or tolerance of animals to the toxicity of cyanotoxins. Crustacea, for instance, have survival rates of 100%, even for exposures to concentrations of cyanotoxins like those in bloom episodes [30,128]. Long-term macroinvertebrates exposure to cyanotoxins via feeding may involve bioaccumulation levels higher than cell-free or dissolved exposures, which suggests that different absorption and metabolization pathways likely exist [30,122,129]. However, another fact is that the macroinvertebrates that cohabit with cyanobacteria or are long-term/chronically exposed to cyanotoxins show more tolerance to toxicity than those that have not been exposed [47,130].

Although the tolerance and detoxification capacity of benthic macroinvertebrates are still not completely elucidated, several studies into invertebrates and vertebrates tend to relate it to the enzymatic response against cellular oxidative stress or the activating immunity system [32,102,105,129,131]. The role of glutathione (GSH) in MC detoxification has long been known [132], but the effect of antioxidants as blockers of cyanotoxin accumulation and the metabolic pathways involved in detoxification processes have become particularly interesting in the last decade, regardless of antioxidants being produced naturally by the organism or obtained through diet, such as astaxanthins [31,32,133]. However, the detoxification process against cyanotoxins could be more complex, because transcriptomic studies have shown that exposure to these toxins triggers the activation of about 44 immune- and redox-related genes associated with metabolic detoxification phases I and II [32]. Other detoxification ways have also been proposed, such as accelerating intestinal food transit or increasing the bacterial flora that degrades cyanotoxins [31,32]. Although the transfer of toxins between trophic levels in relation to invertebrates is unquestionable [99,101,125], the detoxification capacity observed in freshwater invertebrates is beginning to challenge the established belief that they contribute to the biomagnification of toxins along the food chain [1,29,101]. In fact, even at high concentrations of cyanotoxins, as measured in aquatic consumers of different trophic levels, a meta-analysis based on the biomagnification factor has confirmed biodilution, and not biomagnifications, as the dominant process in aquatic food webs [28,29]. The toxicity transfer of some mayfly species to terrestrial predators, such as bats, has led us to begin taking the aerial phases of hexapods as vectors of toxicity transfer from fresh water to the terrestrial food chain [107,134]. Nevertheless, some stonefly, shore fly and bug adults are capable of eating the cyanobacteria available in terrestrial habitats [83,84,91].

The following Table 1 summarize cyanotoxin producers and main toxins synthesized.

A compilation of relationships between cyanobacteria and benthic macroinvertebrates is shown in Table S1. Taxon names in the papers are maintained (independently of their validity or update).

## 3. Conclusions and Future Directions

On the one hand, benthic macroinvertebrates can feed on cyanobacteria, even toxic strains, and are able to survive, grow or complete their life cycle, even by bioaccumulating toxins. On the other hand, the cyanobacteria species or strains that are considered nontoxic can be as harmful to, or are much more harmful than, macroinvertebrates than toxic ones, which suggests that there are other still unknown and potentially toxic compounds. Despite known or unknown toxicity, some macroinvertebrates use cyanobacteria as their main food source, and even choose to live near, on, or in them. Therefore, toxicity should not be the determining factor of the biological relationships between these organisms. The tolerance acquired by benthic macroinvertebrates after millions of years of cohabitation may possibly hold the answer, but the dispersion of currently available data is so wide that it only allows speculation. Perhaps new studies that focus more on the biological synergies of these organisms, rather than on antagonisms, are needed to clear up this mystery.

Exposure of macroinvertebrates to cyanobacteria toxicity can have different short- or long-term effects depending on the way in which it occurs. It has been proven that survival, growth, completing the life cycle, bioaccumulation and behavior can differ if cyanotoxins are obtained through diet or if they are cell-free in water. Toxic effects tend to be generally more lethal or intense with cell-free exposure than when obtained from food. The degree of tolerance might also be responsible for this heterogeneity, and perhaps it can be explained by the existence of different metabolic pathways that manage each exposure type.

Detoxification processes will also be a key factor in macroinvertebrates’ response to cyanobacteria toxicity. These processes are being verified in more organisms, with the activation of the main antioxidant enzymes being the focus of attention. However, it is being shown that detoxification may involve a larger number of metabolic processes related not only to antioxidant machinery but also to the immune system itself. This kind of study will be decisive for unraveling what promotes tolerance in benthic macroinvertebrates, especially as it is known in organisms with a close relationship to cyanobacteria, such as those with a mutualistic relationship.

## Figures and Tables

**Table 1:** Main benthic cyanotoxin producers and main toxic compounds they produce.

Taxa	MCs	ATX	STX	CYN	NODs
*Anabaena*	[2,19,38,40,41,43,46,135]	[19,22,40,41,43,135]	[1,2,22,38,135]	[19,38,135]	-
*Arthrospira*	[135]	[135]	-	-	-
*Calothrix*	[17]	-	-	-	[2]
*Cyanomargarita*	[136]	-	-	-	-
*Cylindrospermum*	[19]	[38,135]	[38,137]	-	-
*Dactylothamnos*	-	-	[136]	-	-
*Fischerella*	[2,19]	-	-	-	-
*Geitlerinema*	[2,18,33,38,44,136]	[2,33,38]	[2,38,137]	-	-
*Gloeotrichia*	[33,38,135]	-	-	-	-
*Hapalosiphon*	[135]	-	-	-	-
*Kamptonema*	-	[136]	-	-	-
*Leptolyngbya*	[18,28,136]	-	-	-	[2]
*Lyngbya*	[2,17,22]	-	[1,22,38,93,135]	[19,38,93]	-
*Microcoleus*	[2,17,38,42,46]	[2,28,38,42,46,56,136]	[38,42]	[38,42]	[2]
*Microseira*	[2]	-	[2]	[2]	-
*Nodularia*	-	-	-	-	[19,135]
*Nostoc*	[2,19,33,38,42,93,135]	[38,42]	[42]	[42]	[2,38,135]
*Oscillatoria*	[1,2,17,18,33,38,42,44,89,93,135]	[19,22,38,42,135,136]	-	[38,42]	-
*Phormidium*	[2,15,16,17,18,33,40,44,51,93,135]	[2,40,56]	[22]	[2]	[2]
*Plectonema*	[2,135]	-	-	-	-
*Pseudanabaena*	[33,53]	-	-	-	-
*Rivularia*	[16,17,18,19,33,62,135]	-	-	-	-
*Schizothrix*	[33]	-	-	-	-
*Scytonema*	[2,33,38,44]	-	[2,22,38,93,136]	-	-
*Tolypothrix*	[16,17,39]	-	-	-	-
*Trichormus*	[38]	-	-	-	-
*Tychonema*	-	[2,56,136]	-	-	-
*Westiellopsis*	[2]	-	-	-	-
*Wollea*	[42]	-	[42]	[42]	-

## Data Availability

The raw data supporting the conclusions of this article will be made available by the author on request.

## Supplementary Materials

The following supporting information can be downloaded at https://www.mdpi.com/article/10.3390/toxins16040190/s1. Table S1: Compilation of data regarding the relationships between cyanobacteria and benthic macroinvertebrates. References [138,139,140,141,142,143,144,145,146,147,148,149,150,151,152,153,154,155,156,157,158,159,160,161,162,163,164,165,166,167,168,169,170,171,172,173,174,175,176,177,178,179,180,181,182,183,184,185,186,187,188,189,190,191,192,193,194,195,196,197,198,199,200,201,202,203,204,205,206,207,208,209,210,211,212,213,214,215,216,217,218,219,220,221,222,223,224,225,226,227,228,229,230,231,232,233,234,235,236,237,238,239,240,241,242,243,244,245,246,247,248,249,250,251,252,253,254,255,256,257,258,259] are cited in Table S1.

## References

[1] Ferrão-Filho A.S., Kozlowsky-Suzuki B. (2011). Cyanotoxins: Bioaccumulation and effects on aquatic animals. Mar. Drugs.

[2] Wood S.A., Kelly L.T., Bouma-Gregson K., Humbert J.-F., Lauginghouse IV H.D., Lazorchak J., McAllister T.G., McQueen A., Pokrzywinski K., Puddick J. (2020). Toxic benthic freshwater cyanobacterial proliferations: Challenges and solutions for enhancing knowledge and improving monitoring and mitigation. Freshw. Biol..

[3] Chorus I., Fastner J., Welker M. (2021). Cyanobacteria and cyanotoxins in a changing environment: Concepts, controversies, challenges. Water.

[4] Waters M.N., Brenner M., Curtis J.H., Romero-Olivad C.S., Dixd M., Canoe M. (2021). Harmful algal blooms and cyanotoxins in Lake Amatitlán, Guatemala, coincided with ancient Maya occupation in the watershed. Proc. Natl. Acad. Sci. USA.

[5] Codd G.A. (1995). Cyanobacterial toxins: Occurrence, properties, and biological significance. Water Sci. Technol..

[6] Francis G. (1878). Poisonous Australian Lake. Nature.

[7] Codd G.A., Plinski M., Surosz W., Hutson J., Fallowfield H.J. (2015). Publication in 1672 of animal deaths at the Tuchomskie Lake, northern Poland and a likely role of cyanobacterial blooms. Toxicon.

[8] Huisman J., Codd G.A., Paerl H.W., Ibelings B.W., Verspagen J.M.H., Visser P.M. (2018). Cyanobacterial blooms. Nat. Rev. Microbiol..

[9] Whitton B.A. (2012). Ecology of Cyanobacteria II: Their Diversity in Space and Time.

[10] Usher K.M., Bergman B., Raven J.A. (2007). Exploring Cyanobacterial Mutualisms. Annu. Rev. Ecol. Evol. Syst..

[11] Sabater S., Guasch H., Romaní A., Muñoz I. (2000). Stromatolitic communities in Mediterranean streams: Adaptations to a changing environment. Biodivers. Conserv..

[12] Waters M.N. (2016). A 4700-year history of cyanobacteria toxin production in a shallow subtropical lake. Ecosystems.

[13] Murray S.A., Mihali T.K., Neilan B.A. (2011). Extraordinary conservation, gene loss, and positive selection in the evolution of an ancient neurotoxin. Mol. Biol. Evol..

[14] Rantala A., Fewer D.P., Hisbergues M., Rouhiainen L., Vaitomaa J., Börner T., Sivonen K. (2004). Phylogenetic evidence for the early evolution of microcystin synthesis. Proc. Natl. Acad. Sci. USA.

[15] Mez K., Beattie K., Codd G.A., Hanselmann K., Hauser B., Naegeli H., Preisig H. (1997). Identification of a microcystin in benthic cyanobacteria linked to cattle deaths on alpine pastures in Switzerland. Eur. J. Phycol..

[16] Aboal M., Puig M.A., Asencio A.D. (2005). Production of microcystins in calcareous Mediterranean streams: The Alhárabe River, Segura River basin in South-East Spain. J. Appl. Phycol..

[17] Aboal M., Puig M.A. (2005). Intracellular and dissolved microcystin in reservoirs of the river Segura basin, Murcia, SE Spain. Toxicon.

[18] Hurtado I., Aboal M., Zafra E., Campillo D. (2008). Significance of microcystin production by benthic communities in water treatment systems of arid zones. Water Res..

[19] Filatova D., Picardo M., Núñez O., Farré M. (2020). Analysis, levels and seasonal variation of cyanotoxins in freshwater ecosystems. Trends Environ. Anal. Chem..

[20] De la Cruz A.A., Hiskia A., Kaloudos T., Chernoff N., Hill D., Antoniou M.G., He X., Loftin K., O’Shea K., Zhao C. (2013). A review on the cylindrospermopsin: The global occurrence, detection, toxicity and degradation of a potent cyanotoxin. Environ. Sci. Process. Impacts.

[21] Aráoz R., Molgó J., Tandeu de Marsac N. (2010). Neurotoxic cyanobacterial toxins. Toxicon.

[22] Christensen V.G., Khan E. (2020). Freshwater neurotoxins and concerns for human, animal, and ecosystem health: A review of anatoxin-a and saxitoxin. Sci. Total Environ..

[23] Koksharova O.A., Safronova N.A. (2022). Non-proteinogenic amino acid *ß-N*-methylamino-*L*-alanine (BMAA): Bioactivity and Ecological significance. Toxins.

[24] Leao P.N., Pereira A.R., Liu W.-T., Ng J., Pevzner P.A., Dorrestein P.C., König G.M., Vasconcelos V.M., Gerwick W.H. (2010). Synergistic allelochemicals from a freshwater cyanobacterium. Proc. Natl. Acad. Sci. USA.

[25] Juhel G., Davenport J., O’Halloran J., Culloty S.C., O’Riordan R.M., James K.F., Furey A., Allis O. (2006). Impacts of microcystins on the feeding behavior and energy balance of zebra mussels, *Dreissenia polymorpha*: A bioenergetics approach. Aquat. Toxicol..

[26] Svircev Z., Chen L., Sántha K., Drobac Backovic D., Susak S., Vulin A., Palanacki Malesevic T., Codd G.A., Meriluoto J. (2022). A review and assessment of cyanobacterial toxins as cardiovascular hazards. Arch. Toxicol..

[27] Polyak Y.M., Polyak M.S. (2022). The role of cyanotoxins in human and animal pathology (a review). J. Microbiol. Epidemiol. Immunol..

[28] Ibelings B.W., Bruning K., Junge J., Wolfstein K., Dionisio L.M., Postma J., Burger T. (2005). Distribution of microcystins in a lake food web: No evidence for biomagnification. Microb. Ecol..

[29] Kozlowsky-Suzuki B., Wilson A., Ferrão-Filho A.S. (2012). Biomagnification or biodilution of microcystins in aquatic food webs? Meta-analyse of laboratory and field studies. Harmful Algae.

[30] Clearwater S.J., Wood S.A., Phillips N.R., Parkyn S.M., Van Ginkel R., Thompson K.J. (2012). Toxicity Thresholds for Juvenile Freshwater Mussels *Echyridella menziesii* and Crayfish *Paranephrops planifrons*, after Acute or Chronic Exposure to *Microcystis* sp. Environ. Toxicol..

[31] Cai S., Shu Y., Tian C., Wang C., Fang T., Xiao B., Wu X. (2022). Effects of chronic exposure to microcystin-LR on life-history traits, intestinal microbiota and transcriptomic responses in *Chironomus pallidivittatus*. Sci. Total Environ..

[32] Zhang Y., Li Z., Kholodkevich S., Sharov A., Feng Y., Ren N., Sun K. (2020). Microcystin-LR-induced changes of hepatopancreatic transcriptome, intestinal microbiota, and histopathology of freshwater crayfish (*Procambarus clarkii*). Sci. Total Environ..

[33] Cantoral E.A., Asencio A.D., Aboal M. (2017). Are we underestimating benthic cyanotoxins? Extensive sampling results from Spain. Toxins.

[34] Falfushynska H., Kasianchuk N., Siemens E., Hemao E., Rzymski P. (2023). A Review of Common Cyanotoxins and Their Effects on Fish. Toxics.

[35] Australian Drinking Water (2011). Guidelines Paper 6 National Water Quality Management Strategy.

[36] Guidelines for Canadian Drinking Water Quality: Guideline Technical Document-Cyanobacterial Toxins. Health Canada, Government Canada. https://www.canada.ca/en/health-canada/services/publications/healthy-living/guidelines-canadian-drinking-water-quality-guideline-technical-document-cyanobacterial-toxins-document.html.

[37] Oehrle S.A., Southwell B., Westrick J. (2010). Detection of various freshwater cyanobacterial toxins using ultra-performance liquid chromatography tandem mass spectrometry. Toxicon.

[38] (2022). Benthic Cyanobacteria and Cyanotoxin Monitoring in Northern California Rivers, 2016–2019.

[39] Bouma-Gregson K. (2017). The Ecology of Benthic Toxigenic *Anabaena* and *Phormidium* (Cyanobacteria) in the Eal River, California. Ph.D. Thesis.

[40] Bouma-Gregson K., Kudela R.M., Power M.E. (2018). Widespread anatoxin-a detection in benthic cyanobacterial mats throughout a river network. PLoS ONE.

[41] Bouma-Gregson K., Power M.E., Bormans M. (2017). Rise and fall of toxic benthic freshwater cyanobacteria (*Anabaena* spp.) in the Eel River: Buoyancy and dispersal. Harmful Algae.

[42] Carpenter K. (2021). Benthic periphyton as a source of cyanotoxins in three Oregon rivers used for municipal drinking water supply. Protecting Drinking Water from Cyano-HAB Impacts in the Willamette Basin, Willamette Basin Partners’ Workshop.

[43] Kelly L.T. (2019). Toxic Benthic Cyanobacteria (*Microcoleus autumnalis*): Genetic Structure and Ecological Effects. Ph.D. Thesis.

[44] Valdor R., Aboal M. (2007). Effects of living cyanobacteria, cyanobacterial extracts and pure microcystins on growth and ultrastructure of microalgae and bacteria. Toxicon.

[45] Pietsch C., Wiegand C., Amé M.V., Nicklisch A., Wunderlin D., Pflugmacher S. (2001). The effects of a cyanobacterial crude extract on different aquatic organisms: Evidence for cyanobacterial toxin modulating factors. Environ. Toxicol..

[46] Kelly L.T., Bouma-Gregson K., Puddick J., Fadness R., Ryan K.G., Davis T.W., Wood S.A. (2019). Multiple cyanotoxin congeners produced by sub-dominant cyanobacterial taxa in riverine cyanobacterial and algal mats. PLoS ONE.

[47] Aboal M., Puig M.A., Ríos H., López-Jiménez E. (2000). Relationship between macroinvertebrate diversity and toxicity of cyanophyceae (Cyanobacteria) in some streams from Eastern Spain. Verh. Int. Ver. Limnol..

[48] Efting A.A., Snow D.D., Fritz S.C. (2011). Cyanobacteria and microcystin in the Nebraska (USA) Dand Hills Lakes before and after modern agriculture. J. Paleolimnol..

[49] Erratt K., Creed I.F., Favot E.J., Todoran I., Tai V., Smol J.P., Trick C.G. (2021). Paleolimnological evidence reveals climate-related preeminence of cyanobacteria in a temperate meromictic lake. Can. J. Fish. Aquat. Sci..

[50] Zastepa A., Taranu Z.E., Kimpe L.E., Blais J.M., Gregory-Eaves I., Zurawell R.W., Pick F.R. (2017). Reconstructing a long-term record of microcystins from the analysis of lake sediments. Sci. Total Environ..

[51] Wood S.A., Heath M.W., Holland P.T., Munday R., McGregor G.B., Ryan K.G. (2010). Identification of a benthic microcystin-producing filamentous cyanobacterium (Oscillatoriales) associated with a dog poisoning in New Zealand. Toxicon.

[52] Stal L.J., Krumbein W.E. (1987). Temporal separation of nitrogen fixation and photosynthesis in the filamentous, non-heterocystous cytanobacterium *Oscillatoria* sp. Arch. Microbiol..

[53] Maryan P.S., Eady R.R., Chaplin A.E., Gallon J.R. (1986). Nitrogen fixation by *Gloeothece* sp. PCC6909: Respiration and not photosynthesis supports nitrogenase activity in the light. Microbiology.

[54] Dolman A.M., Rücker J., Pick F.R., Fastner J., Rohrlack T., Mischke U., Wiedner C. (2012). Cyanobacteria and Cyanotoxins: The influence of Nitrogen versus Phosphorus. PLoS ONE.

[55] Moreno I.M., Pereira P., Franca S., Cameán A. (2004). Toxic cyanobacteria strains isolated from blooms in the Guadiana River/southwestern Spain. Biol. Res..

[56] Devlin J.P., Edwards E., Gorham P.R., Hunter N.R., Pike R.K., Stavric B. (1977). Anatoxin-a, a toxic alkaloid from *Anabaena flos-aquae* NRC-44h. Can. J. Chem..

[57] Carmichael W.W., Jones C.L.A., Mahmood N.A., Theiss W.C. (1985). Algal toxins and water-based diseases. Crit. Rev. Environ. Control..

[58] Cox P.A., Banack S.A., Murch S.J., Rasmussen U., Tien G., Bidigare R.R., Metcalf J.S., Morrison L.F., Codd G.A., Bergman B. (2005). Diverse taxa of cyanobacteria produce B-N-methylamino-L-alanine, a neurotoxic amino acid. Proc. Natl. Acad. Sci. USA.

[59] Ibelings B.W., Kurmayer R., Azevedo S.M.F.O., Wood S.A., Chorus I., Welker M., Chorus I., Welker M. (2021). Understanding the occurrence of cyanobacteria and cyanotoxins (Chapter 4). Toxic Cyanobacteria in Water: A Guide to Their Public Health Consequences, Monitoring and Management.

[60] Kotak B.G., Zurawell R.W. (2007). Cyanobacterial toxins in Canadian freshwaters: A review. Lake Reserv. Manag..

[61] Young F.M., Morrison L.F., James J., Codd G.A. (2008). Quantification and localization of microcystins in colonies of a laboratory strains of *Microcystis* (Cyanobacteria) using immunological methods. Eur. J. Phycol..

[62] Marco S., Aboal M., Chaves-Pozo E., Mulero I., García-Ayala A. (2011). Immunolocalization of microcystins in colonies of the cyanobacterium *Rivularia* in calcareous streams. Mar. Freshw. Res..

[63] Giussani V., Sbrana F., Asnaghi V., Vassalli M., Faimali M., Casabianca S., Penna A., Ciminiello P., Dell’Aversano C., Tartaglione L. (2015). Active role of the mucilage in the toxicity mechanism of the harmful benthic dinoflagellate *Ostreopsis* cf. ovata. Harmful Algae.

[64] Whitton B.A., Grainger S.L.J., Hawley G.R.W., Simon J.W. (1991). Cell-bound and extracellular phosphatase activities of cyanobacterial isolates. Microb. Ecol..

[65] Aboal M., García-Fernández M.E., Roldán M., Whitton B.A. (2014). Ecology, morphology, and physiology of *Chroothece richteriana* (Rhodophyta, Stylonemataceae) in the highly calcareous Río Chícamo, south-east Spain. Eur. J. Phycol..

[66] Komarek J. (2016). A Polyphasic approach for the taxonomy of cyanobacteria: Principles and applications. Eur. J. Phycol..

[67] Guiry M.D., Guiry G.M. (2023). Algaebase. World-Wide Electronic Publication.

[68] Kaštovský J. (2024). Welcome to the jungle!: An overview of modern taxonomy of cyanobacteria. Hydrobiologia.

[69] Trout-Haney J.V., Ritger A., Cottingham K.L. (2021). Benthic cyanobacteria of the genus *Nostoc* are a source of microcystins in Greenlandic lakes and ponds. Freshw. Biol..

[70] Aboal M., Puig M.A., Mateo P., Perona E. (2002). Implications of cyanophyte toxicity on biological monitoring of calcareous streams in North-East Spain. J. Appl. Phycol..

[71] Bownik A. (2016). Harmful algae: Effects of cyanobacterial cyclic peptides on aquatic invertebrates—A short review. Toxicon.

[72] Fadel A., Guerrieri F., Pincebourde S. (2023). The functional relationship between aquatic insects and cyanobacteria: A systematic literature review reveals major knowledge gaps. Total Environ. Res. Themes.

[73] Dudley T.L., Cooper S.D., Hemphill N. (1986). Effects of Macroalgae on a Stream Invertebrate Community. J. N. Am. Benthol. Soc..

[74] Ferrão-Filho A.S. (2009). Bioacumulación de cianotoxinas y sus efectos en organismos acuáticos (Bioacumulaçao de cianotoxinas e seus efeitos em organismos aquáticos). Oecol. Bras..

[75] Abdallah M.F., Rajkovic A. (2021). Cyanotoxins and Food Contamination in Developing Countries: Review of Their Types, Toxicity, Analysis, Occurrence and Mitigation Strategies. Toxins.

[76] Briland R.D., Stone J.P., Manubolu M., Leeb J., Ludsin S.A. (2020). Cyanobacterial blooms modify food web structure and interactions in western Lake Erie. Harmful Algae.

[77] Aboal M., Belando M.D., Ubero N., González-Silvera D., López-Jiménez J.A. (2022). Photoautotrophs and macroinvertebrate trophic relations in calcareous semiarid streams: The role of Cyanobacteria. Sci. Total Environ..

[78] Gérard C., Lance E. (2019). Decline of freshwater gastropods exposed to recurrent interacting stressors implying cyanobacterial proliferations and droughts. Aquat. Ecol..

[79] Hart D.D. (1985). Grazing insects mediate algal interactions in a stream benthic community. Oikos.

[80] Scott J.T., Marcarelli A.M., Whitton B.A. (2012). Cyanobacteria in Freshwater Benthic Environments. Ecology of Cyanobacteria II: Their Diversity in Space and Time.

[81] Berezina N.A., Tiunov A.V., Tsurikov S.M., Kurbatova S.A., Korneva L.G., Makarova O.S., Bykova S.N. (2021). Cyanobacteria as a food source for invertebrates: Results of a model experiment. Russ. J. Ecol..

[82] Chen L., Giesy J.P., Adamovsky O., Svirčev Z., Meriluoto J., Codd G.A., Mijovic B., Shi T., Tuo X., Li S.-C. (2021). Challenges of using blooms of *Microcystis* spp. in animal feeds: A comprehensive review of nutritional, toxicological and microbial health evaluation. Sci. Total Environ..

[83] Krivosheina M. (2008). On insect feeding on cyanobacteria. Paleontol. J..

[84] Hädicke C.W., Rédei D., Kment P. (2017). The diversity of feeding habits recorded for water boatmen (Heteroptera: Corixoidea) world-wide with implications for evaluating information on the diet of aquatic insects. Eur. J. Entomol..

[85] Ashe P., Murray D.A., Murray D.A. (1980). *Nostococladius*, a new subgenus of *Cricotopus* (Diptera: Chironomidae). Chironomidae.

[86] Foote B.A. (1993). Biology of *Hyadina albovenosa* (Diptera, Ephydridae), a consumer of cyanobacteria. Proc. Entomol. Soc. Wash..

[87] Hollows J.W., Townsend C.R., Collier K.J. (2002). Diet of the crayfish *Paranephrops zealandicus* in bush and pasture streams: Insights from stable isotopes and stomach analysis. N. Z. J. Mar. Freshw. Res..

[88] Frouz J., Ali A., Lobinske R.J. (2004). Algal food selection and digestion by larvae of the pestiferous chironomid *Chironomus crassicaudatus* under laboratory conditions. J. Am. Mosq. Control Assoc..

[89] Frouz J., Ali A., Lobinske R.J. (2004). Laboratory Evaluation of Six Algal Species for Larval Nutritional Suitability of the Pestiferous Midge *Glyptotendipes paripes* (Diptera: Chironomidae). J. Econ. Entomol..

[90] Toporowska M., Pawlik-Skowronska B., Kalinowska R. (2014). Accumulation and effects of cyanobacterial microcystins and anatoxin-a on benthic larvae of *Chironomus* spp. (Diptera: Chironomidae). Eur. J. Entomol..

[91] Tierno de Figueroa J.M., López-Rodríguez M.J. (2019). Trophic ecology of Plecoptera (Insecta): A review. Eur. Zool. J..

[92] Aydin G.B., Öterler B., Çamur Elipek B., Güher H. (2021). The Comparative Gut Content Analysis of Some Chironomidae Larvae Living in the Freshwaters at Northern Thrace Region of Turkey. J. Limnol. Freshw. Fisheries Res..

[93] Brock E.M. (1960). Mutualism between the midge *Cricotopus* and the alga *Nostoc*. Ecology.

[94] Quiblier C., Wood S., Echenique-Subiabre I., Heath M., Villeneuve A., Humbert J.-F. (2013). A review of current knowledge on toxic benthic freshwater cyanobacteria-Ecology, toxin production and risk management. Water Res..

[95] Dionne K., Dufresne F., Nozais C. (2016). Variation in δ13C and δ15N trophic enrichment factors among *Hyalella azteca* amphipods from different lakes. Hydrobiologia.

[96] Ward A.K., Dahm C.N., Cummins K.W. (1985). *Nostoc* (Cyanophyta) productivity in Oregon stream ecosystems: Invertebrate influences and differences between morphologycal types. J. Phycol..

[97] Xue Q., Su X., Steinman A.D., Cai Y., Zhao Y., Xie L. (2016). Accumulation of microcystins in a dominant Chironomid Larvae (*Tanypus chinensis*) of a large, shallow and eutrophic Chinese lake, Lake Taihu. Sci. Rep..

[98] Salas M., Dudgeon D. (2001). Stable-isotope determination of mayfly (Insecta: Ephemeroptera) food sources in three tropical Asian streams. Arch. Hydrobiol..

[99] Chen J., Xie P. (2005). Tissue distributions and seasonal dynamics of the hepatotoxic microcystins-LR and -RR in two freshwater shrimps, *Palaemon modestus* and *Macrobrachium nipponensis*, from a large shallow, eutrophic lake of the subtropical China. Toxicon.

[100] Goedkoop W., Kerblom N.A., Demandt M.H. (2006). Trophic fractionation of carbon and nitrogen stable isotopes in *Chironomus riparius* reared on food of aquatic and terrestrial origin. Freshw. Biol..

[101] Liu L.P., Su X.M., Chen T.Y., Li K., Zhan J., Egna H., Diana J. (2016). Evidence of rapid transfer and bioaccumulation of Microcystin-LR poses potential risk to freshwater prawn *Macrobrachium rosenbergii* (de Man). Aquac. Res..

[102] Stanković N., Kostić I., Jovanović B., Savić-Zdravković D., Matić S., Bašić J., Cvetković T., Simeunović J., Milošević D. (2020). Can phytoplankton blooming be harmful to benthic organisms? The toxic influence of *Anabaena* sp. and *Chlorella* sp. on *Chironomus riparius* larvae. Sci. Total Environ..

[103] Smith J.L., Boyer G.L., Mills E., Schulz K.L. (2008). Toxicity of Microcystin-LR, a Cyanobacterial Toxin, to Multiple Life Stages of the Burrowing Mayfly, *Hexagenia,* and Possible Implications for Recruitment. Environ. Toxicol..

[104] Gaget V., Almuhtaram H., Kibuye F., Hobson P., Zamyadi A., Wert E., Brookes J.D. (2022). Benthic cyanobacteria: A utility-centred field study. Harmful Algae.

[105] Babica P., Kohoutek J., Bláha L., Adamovsky O., Maršálek B. (2006). Evaluation of extraction approaches linked to ELISA and HPLC for analyses of microcystin-LR, -RR and -YR in freshwater sediments with different organic material contents. Anal. Bioanal. Chem..

[106] Preece E.P., Hobbs W., Hardy F.J., O’Garro L., Frame E., Sweeney F. (2021). Prevalence and persistence of microcystin in shoreline lake sediments and porewater, and associated potential for human health risk. Chemosphere.

[107] Woller-Skar M.M., Russell A.L., Gaskill J.A., Luttenton M.R. (2020). Microcystin in multiple life stages of *Hexagenia limbata*, with implications for toxin transfer. J. Gt. Lakes Res..

[108] Stepanian P.M., Entrekin S.A., Wainwright C.E., Mirkovic D., Tank J.L., Kelly J.F. (2020). Declines in an abundant aquatic insect, the burrowing mayfly, across major North American waterways. Proc. Natl. Acad. Sci. USA.

[109] He Q., Wang W., Xu Q., Liu Z., Teng J., Yan H., Liu X. (2022). Microcystins in Water: Detection, Microbial Degradation Strategies, and Mechanisms. Int. J. Environ. Res. Public Health.

[110] Salter C., Westrick J.A., Chaganti S.R., Birbeck J.A., Peraino N.J., Weisener C.G. (2023). Elucidating microbial mechanisms of microcystin-LR degradation in Lake Erie beach sand through metabolomics and metatranscriptomics. Water Res..

[111] Dziga D., Wasylewski M., Wladyka B., Nybom S., Meriluoto J. (2013). Microbial Degradation of Microcystins. Chem. Res. Toxicol..

[112] Massey I.Y., Yang F. (2020). A Mini Review on Microcystins and Bacterial Degradation. Toxins.

[113] Jacobus L.M., McCafferty W.P. (2004). Contribution to the morphology and descriptive biology of *Caurinella idahoensis* (Ephemeroptera, Ephemerellidae). West. N. Am. Nat..

[114] Tachibana S. (2022). A new species, *Cricotopus cataractaenostocicola*, living in a cyanobacterial colony on vertical rocky substrates with trickling water film in Japan (Diptera: Chironomidae). Zootaxa.

[115] Dodds W., Marra J. (1989). Behaviors of the midge, *Cricotopus* (Diptera: Chironomidae) related to mutualism with *Nostoc parmelioides* (Cyanobacteria). Aquat. Insects.

[116] Langton P.H., Casas J. (1999). Changes in chironomid assemblage composition in two Mediterranean mountain streams over a period of extreme hydrological conditions. Hydrobiologia.

[117] Sabater S., Muñoz I. (2000). *Nostoc verrucosum* (Cyanobacteria) colonized by a chironomid larva in a Mediterranean stream. J. Phycol..

[118] Sanz-Montero M.E., Calvo J.P., García del Cura M.A., Ornosa C., Outerelo R., Rodríguez-Aranda J.P. (2013). The rise of the diptera-microbial mat interactions during the Cenozoic: Consequences for the sedimentary record of saline lakes. Terra Nova.

[119] Hägele D., Leinfelder R., Grau J., Burmeister E.-G., Struck U. (2006). Oncoids from the river Alz (southern Germany): Tiny ecosystems in a phosphorus-limited environment. Palaeogeogr. Palaeoclimatol. Palaeoecol..

[120] Liarte S., Ubero-Pascal N., García-Ayala A., Puig M.A. (2014). Histological effects and localization of dissolved microcystins LR and LW in the mayfly *Ecdyonurus angelieri* Thomas (Insecta, Ephemeroptera). Toxicon.

[121] Zhang Y., Zhuang H., Yang H., Xue W., Wang L., Wei W. (2019). Microcystin-LR disturbs testicular development of giant freshwater prawn *Macrobrachium rosenbergii*. Chemosphere.

[122] Saker M.L., Eaglesham G.K. (1999). The accumulation of cylindrospermopsin from the cyanobacterium *Cylindrospermopsis raciborskii* in tissues of the Redclaw crayfish *Cherax quadricarinatus*. Toxicon.

[123] Vasconcelos V., Oliveira S., Teles F.O. (2001). Impact of a toxic and a non-toxic strain of *Microcystis aeruginosa* on the crayfish *Procambarus clarkii*. Toxicon.

[124] Chen J., Xie P. (2008). Accumulation of hepatotoxic microcystins in freshwater mussels, aquatic insect larvae and oligochaetes in a large, shallow eutrophic lake (Lake Chaohu) of subtropical China. Fresenius Environ. Bull..

[125] Lance E., Lepoutre A., Savar V., Robert E., Bormans M., Amzil Z. (2021). In situ use of bivalves and passive samplers to reveal water contamination by microcystins along a freshwater-marine continuum in France. Water Res..

[126] Singh P., Snokhousová J., Saraf A., Suradkar A., Elster J. (2020). Phylogenetic evaluation of the genus *Nostoc* and description of *Nostoc neudorfense* sp. nov., from the Czech Republic. Int. J. Syst. Evol. Microbiol..

[127] Delaney J.M., Wilkins R.M. (1995). Toxicity of microcystin-LR, isolated from *Microcystis* aeruginosa, against various insect species. Toxicon.

[128] Galanti L.N., Amé M.V., Wunderlin D.A. (2013). Accumulation and detoxification dynamic of cyanotoxins in the freshwater shrimp *Palaemonetes argentinus*. Harmful Algae.

[129] Liu Y., Yang M., Zheng L., Nguyen H., Ni L., Song S., Sui Y. (2020). Antioxidant responses of triangle sail mussel *Hyriopsis cumingii* exposed to toxic *Microcystis* aeruginosa and thermal stress. Sci. Total Environ..

[130] Mills D.H., Wyatt J.T. (1974). Ostracod Reactions to Non-Toxic and Toxic Algae. Oecologia.

[131] Krosch M.N., Bryant L.M., Vink S. (2017). Differential gene expression of Australian *Cricotopus draysoni* (Diptera: Chironomidae) populations reveals seasonal association in detoxification gene regulation. Sci. Rep..

[132] Plugmacher S., Wiegand C., Oberemm A., Beattie K.A., Krause E., Codd G.A., Steinberg C.E.V. (1998). Identification of an enzymatically formed glutathione conjugate of the cyanobacterial hepatotoxin microcystin-LR: The first step of detoxication. Biochim. Biophys. Acta.

[133] An Z., Zhang Y., Sun L. (2018). Effects of Dietary Astaxanthin Supplementation on Energy Budget and Bioaccumulation in *Procambarus clarkii* (Girard, 1852) Crayfish under Microcystin-LR Stress. Toxins.

[134] Woller-Skar M.M., Jones D.N., Luttenton M.R., Russell A.L. (2015). Microcystin Detected in Little Brown Bats (*Myotis lucifugus*). Am. Midi. Nat..

[135] Pokrzywinski K., Volk K., Wood S., Davis T., Lazorchak J. (2021). Aligning Research and Monitoring Priorities for Benthic Cyanobacteria and Cyanotoxins: A Workshop Summary. Great Lakes Restoration Initiative.

[136] Bauer F., Wolfschlagr I., Gesit J., Fastner J., Wiena Schmalz C., Raeder U. (2023). Occurrence, Distribution and Toxins of Benthic Cyanobacteria in German Lakes. Toxics.

[137] Borges H.L.F., Branco L.H.Z., Martins M.D., Lima C.S., Barbosa P.T., Lira G.A.S.T., Bittencourt-Oliveira M.C., Molica R.J.R. (2015). Cyanotoxins production and phylogeny of benthic cyanobacterial strains isolated from northeast of Brazil. Harmful Algae.

[138] Laurén-Määttä C., Hietala J., Reinikainen M., Walls M. (1995). Do *Microcystis aeruginosa* toxins accumulate in the food web: A laboratory study. Hydrobiologia.

[139] Kawecka B., Kownacki A., Kownacka M. (1978). Food relations between algae and bottom fauna communities in glacial streams. Verh. Int. Ver. Theor. Angew. Limnol..

[140] Foote B.A. (1983). Biology and immature stages of *Nostima approximata* (Diptera, Ephydridae), a grazer of the blue-green algal genus *Oscillatoria*. Proc. Entomol. Soc. Wash..

[141] Kaczorowska A., Kornijów R. (2012). Palaeoecological evidence for changes over the past 200 years in chironomid communities of a shallow lake exposed to cyanobacterial toxins. Aquat. Ecol..

[142] Kajac Z., Warda J. (1968). Feeding of benthic non-predatory Chironomidae in lakes. Ann. Zool. Fenn..

[143] Oberholster P.J., Botha A.M., Ashton P.J. (2009). The influence of a toxic cyanobacterial bloom and water hydrology on algal populations and macroinvertebrate abundance in the upper littoral zone of Lake Krugersdrift, South Africa. Ecotoxicology.

[144] Imada Y. (2020). A novel leaf-rolling chironomid, *Eukiefferiella endobryonia* sp. nov. (Diptera, Chironomidae, Orthocladiinae), highlights the diversity of underwater chironomid tube structures. ZooKeys.

[145] Tourville Poirier A.M., Cattaneo A., Hudon C. (2010). Benthic cyanobacteria and filamentous chlorophytes affect macroinvertebrate assemblages in a large fluvial lake. J. N. Am. Benthol. Soc..

[146] Kornijów R., Markiyanova M., Lange E. (2019). Feeding by two closely related species of *Chironomus* (Diptera: Chironomidae) in south Baltic lagoons, with implications for competitive interactions and resource partitioning. Aquat. Ecol..

[147] Ali A. (1990). Seasonal changes of larval food and feeding of *Chironomus crassicaudatus* (Diptera: Chironomidae) in a subtropical lake. J. Am. Mosq. Control Assoc..

[148] Ali A., Frouz J., Lobinske R.J. (2022). Spatio-temporal effects of selected physico-chemical variables of water, algae and sediment chemistry on the larval community of nuisance Chironomidae (Diptera) in a natural and a man-made lake in central Florida. Hydrobiologia.

[149] Provost M.W., Branch N. (1959). Food of Chironomid Larvae in Polk County Lakes. Fla. Entomol..

[150] Anderson B., Voorhees J., Phillips B., Fadness R., Stancheva R., Nichols J., Orr D., Wood S.A. (2018). Extracts from benthic anatoxin-producing *Phormidium* are toxic to 3 macroinvertebrate taxa at environmentally relevant concentrations. Environ. Toxicol. Chem..

[151] Cai S., Jia Y., Donde O.O., Wang Z., Zhang J., Fang T., Xiao B., Wu X. (2021). Effects of microcystin-producing and non-microcystin-producing *Microcystis* on the behavior and life history traits of *Chironomus pallidivittatus*. Environ. Pollut..

[152] Stanković N., Jovanović B., Kostić Kokić I., Stojković Piperac M., Simeunovć J., Jakimov D., Dimkić I., Milošević D. (2022). Toxic effects of a cyanobacterial strain on *Chironomus riparius* larvae in a multistress environment. Aquat. Toxicol..

[153] Beck S., Wu M. (2021). Effects of *Microcystis aeruginosa* on New Jersey Aquatic Benthic Macroinvertebrates. Adv. Microbiol..

[154] Szczerkowska-Majchrzak E., Jarosiewicz M. (2020). A comparative study of the oxidative system in Chironomidae larvae with contrasting feeding strategies. Eur. Zool. J..

[155] Wirth W.W. (1957). The species of *Cricotopus* midges living in the blue-green alga *Nostoc* in California. Pan.-Pac. Entomol..

[156] Boesel M.V. (1983). A review of the genus *Cricotopus* in Ohio, with a key to adults of species of the Northeastern United States (Diptera, Chironomidae). Ohio J. Sci..

[157] Tarkowska-Kukuryk M. (2013). Periphytic algae as food source for grazing chironomids in a shallow phytoplankton-dominated lake. Limnologica.

[158] Cai S., Wu H., Hong P., Donde O.O., Wang C., Fang T., Xiao B., Wu X. (2021). Bioflocculation effect of *Glyptotendipes tokunagai* on different *Microcystis* species: Interactions between secreted silk and extracellular polymeric substances. Chemosphere.

[159] Henriques-Oliveira A.L., Nessimian J.L., Dorvillé L.F.M. (2023). Feeding habits of *Chironomid larvae* (Insecta: Diptera) from a stream in the Floresta da Tijuca, Rio de Janeiro, Brazil. Braz. J. Biol..

[160] Komulaynen S.F. (2006). Diets of Periphytonic Invertebrates in a Small River. Russ. J. Ecol..

[161] Monroe J.B., LeRoy Poff N., Thorp R.A. (2005). Natural history of a retreat-building midge, *Pagastia partica*, in a regulated reach of the upper Colorado River. West. N. Am. Nat..

[162] Yeh C.C., Chuang Y.Y. (1996). Colonization and bionomics of *Forcipomyia taiwana* (Diptera: Ceratopogonidae) in the laboratory. J. Med. Entomol..

[163] Chan K.L., Leroux E.J. (1971). Nine new species of *Forcipomyia* (diptera: Ceratopogonidae) described in all stages. Can. Entomol..

[164] Foote B.A. (1977). Utilization of blue-green algae by larvae of shore flies. Environ. Entomol..

[165] Foote B.A. (1990). Biology and immature stages of *Coenia curvicauda* (Diptera, Ephydridae), a grazer of the blue-green algal genus *Oscillatoria*. Proc. Entomol. Soc. Wash..

[166] Scheiring J.F., Foote B.A. (1973). Habitat distribution of the shore-flies of Northeastern Ohio (Diptera: Ephydridae). Ohio J. Sci..

[167] Brock M.L., Wiegert R.G., Brock T.D. (1969). Feeding by *Paracoenia* and *Ephydra* (Diptera: Ephydridae) on the Microorganisms of Hot Springs. Ecology.

[168] Collins N. (1980). Population ecology of *Ephydra cinerea* Jones (Diptera: Ephydridae), the only benthic metazoan of the Great Salt Lake, USA. Hydrobiologia.

[169] Thier R.W., Foote B.A. (1980). Biology of mude-shore Ephydridae (Diptera). Proc. Entomol. Soc. Wash..

[170] Collins N.C. (1975). Population Biology of a Brine Fly (Diptera: Ephydridae) in the Presence of Abundant Algal Food. Ecology.

[171] Foote B.A. (1981). Biology and immature stages of *Lytogaster excavata*, a grazer of blue-green algae (Diptera, Ephydridae). Proc. Entomol. Soc. Wash..

[172] Wiegert R.G., Mitchell R. (1973). Ecology of Yellowstone thermal effluent systems intersects of blue-green algae, grazing flies (*Paracoenia*, Ephydridae) and water mites (*Partnuniella*, Hydrachnellae). Hydrobiologia.

[173] Collins N.C., Mitchell R., Wiegert R.G. (1976). Functional Analysis of a Thermal Spring Ecosystem, with an Evaluation of the Role of Consumers. Ecology.

[174] Foote B.A. (1981). Biology and immature stages of *Pelina trunctatula*, a consumer of blue-green algae (Diptera, Ephydridae). Proc. Entomol. Soc. Wash..

[175] Connell T.D., Scheiring J.F. (1981). The feeding ecology of the larvae of the shore fly *Scatella picea* (Walker) (Diptera: Ephydridae). Can. J. Zool..

[176] Zack R.S., Foote B.A. (1978). Utilization of algal monocultures by larvae of *Scatella stagnalis*. Environ. Entomol..

[177] Foote B.A. (1982). Biology and immature stages of *Setacera atrovirens*, a grazer of floating algal mats (Diptera, Ephydridae). Proc. Entomol. Soc. Wash..

[178] Berezina N.A., Verbitsky V.B., Sharov A.N., Chernova E.N., Meteleva N.Y., Malysheva O.A. (2020). Biomarkers in bivalve mollusks and amphipods for assessment of effects linked to cyanobacteria and elodea: Mesocosm study. Ecotoxicol. Environ. Saf..

[179] López-Rodríguez M.J., Tierno de Figueroa J.M., Alba-Tercedor J. (2009). Life history of two burrowing aquatic insects in southern Europe: *Leuctra geniculata* (Insecta: Plecoptera) and *Ephemera danica* (Insecta: Ephemeroptera). Aquat. Insects.

[180] Shahmohamadloo R.S., Poirier D.G., Ortiz Almirall X., Bhavsar S.P., Sibley P.K. (2020). Assessing the toxicity of cell-bound microcystins on freshwater pelagic and benthic invertebrates. Ecotoxicol. Environ. Saf..

[181] Jones D.N., Boyer G.L., Lankton J.S., Woller-Skar M.M., Russell A.L. (2022). Are little brown bats (*Myotis lucifugus*) impacted by dietary exposure to microcystin?. Harmful Algae.

[182] Kelly L.T., Puddick J., Ryan K.G., Champeau O., Wood S.A. (2020). An ecotoxicological assessment of the acute toxicity of anatoxin congeners on New Zealand *Deleatidium* species (mayflies). Inland Waters.

[183] Frison T.H. (1929). Fall and Winter Stoneflies, or Plecoptera, of Illinois. Bull. Ill. Nat. Hist. Surv..

[184] Frison T.H. (1935). The Stoneflies, or Plecoptera, of Illinois. Bull. Ill. Nat. Hist. Surv..

[185] Tierno de Figueroa J.M., Sánchez-Ortega A. (2000). Imaginal feeding of twelve nemouroidean stonefly species (Insecta, Plecoptera). Ann. Entomol. Soc. Am..

[186] Tierno de Figueroa J.M., Sezzi E., Fochetti R. (2003). Feeding in the genus *Tyrrhenoleuctra* (Plecoptera; Leuctridae). Boll. Soc. Entomol. Ital..

[187] Tierno de Figueroa J.M., Sánchez-Ortega A. (1999). Imaginal Feeding of Certain *Systellognathan* Stonefly Species (Insecta: Plecoptera). Ann. Entomol. Soc. Am..

[188] Derka T., Tierno de Figueroa J.M., Krno I. (2004). Life Cycle, Feeding and production of *Isoptena serricornis* (PICTET, 1841) (Plecoptera, Chloroperlidae). Internat. Rev. Hydrobiol..

[189] Tierno de Figueroa J.M., Luzón-Ortega J.M., Sánchez-Ortega A. (1998). Imaginal biology of the stonefly *Hemimelaena flaviventris* (Pictet, 1841) (Plecoptera: Perlodidae). Ann. Zool. Fenn..

[190] Graça M.A.S., Callisto M., Barbosa J.E.L., Firmiano K.R., França J., Gonçalves J.F. (2018). Top-down and bottom-up control of epilithic periphyton in a tropical stream. Freshw. Sci..

[191] Hart D.D., Biggs B.J.F., Nikora V.I., Flinders C.A. (2013). Flow effects on periphyton patches and their ecological consequences in a New Zealand river. Freshw. Biol..

[192] Reynolds J.D. (1975). Feeding in corixids (Heteroptera) of small alkaline lakes in central BC. Int. Ver. Theor. Angew. Limnol..

[193] Hungerford H.B. (1917). Food Habits of Corixids. J. N. Y. Entomol. Soc..

[194] Griffith M.E. (1945). The environment, life history and structure of the waterman boatman, *Ramphocorixa acuminata* (Uhler) (Hemiptera, corixidae). Univ. Kans. Sci. Bull..

[195] Sutton M.F. (1951). On the food, feeding mechanism and alimentary canal of Corixidae (Hemiptera, Heteroptera). Proc. Zool. Soc. Lond..

[196] Camacho F.A., Thacker R.W. (2013). Predator cues alter habitat use by the amphipod *Hyalella azteca* (Saussure). Freshw. Sci..

[197] Kim M.S., Kwon J.T., Lee Y., Ha S.Y., Hong S., Yoon S.H., Shin K.H. (2018). Biocontrol of Microcystis aeruginosa bloom using various aquatic organisms by dual stable isotope (^13^C and ^15^N) tracers. Appl. Ecol. Environ. Res..

[198] Miles C.O., Sandvik M., Haande S., Nonga H., Ballot A. (2013). LC-MS analysis with thiol derivatization to differentiate [Dhb(7)]- from [Mdha(7)]-microcystins: Analysis of cyanobacterial blooms, *Planktothrix* cultures and European crayfish from Lake Steinsfjorden, Norway. Environ. Sci. Technol..

[199] Lirås V., Lindberg M., Nyström P., Annadotter H., Lawton L.A., Graf B. (1998). Can ingested cyanobacteria be harmful to the signal crayfish (*Pacifastacus leniusculus*)?. Freshw. Biol..

[200] Wood S.A., Phillips N.R., de Winton M., Gibbs M. (2012). Consumption of benthic cyanobacterial mats and nodularin-R accumulation in freshwater crayfish (*Paranephrops planifrons*) in Lake Tikitapu (Rotorua, New Zealand). Harmful Algae.

[201] Ríos V., Moreno I., Prieto A.I., Puerto M., Gutiérrez-Praena D., Soria-Díaz M.E., Cameán A.M. (2013). Analysis of MC-LR and MC-RR in tissue from freshwater fish (*Tinca tinca*) and crayfish (*Procambarus clarkii*) in tench ponds (Cáceres, Spain) by liquid chromatography-mass spectrometry (LC-MS). Food Chem. Toxicol..

[202] Monakov A.V. (1972). Review of Studies on Feeding of Aquatic Invertebrates Conducted at the Institute of Biology of Inland Waters, Academy of Science USSR. J. Fish. Res. Board Can..

[203] Freitas M., Azevedo J., Carvalho A.P., Campos A., Vasconcelos V. (2014). Effects of storage, processing and proteolytic digestion on microcystin-LR concentration in edible clams. Food Chem. Toxicol..

[204] Silva C., Anselmo A., Macário I.P.E., de Figueiredo D., Gonçalves F.J.M., Pereira J. (2020). The bad against the villain: Suitability of *Corbicula fluminea* as a bioremediation agent towards cyanobacterial blooms. Ecol. Eng..

[205] Downing S., Contardo-Jara V., Pflugmacher S., Downing T.G. (2014). The fate of the cyanobacterial toxin β-N-methylamino-L-alanine in freshwater mussels. Ecotoxicol. Environ. Saf..

[206] Pham T.L., Shimizu K., Kanazawa A., Gao Y., Dao T.S., Utsumi M. (2016). Microcystin accumulation and biochemical responses in the edible clam *Corbicula leana* P. exposed to cyanobacterial crude extract. J. Environ. Sci..

[207] Ozawa K., Yokoyama A., Ishikawa K., Kumagai M., Watanabe M.F., Park H.D. (2003). Accumulation and depuration of microcystin produced by the cyanobacterium *Microcystis* in a freshwater snail. Limnology.

[208] Gaskill J.A., Woller-Skar M.M. (2018). Do invasive dreissenid mussels influence spatial and temporal patterns of toxic *Microcystis aeruginosa* in a low-nutrient Michigan lake?. Lake Reserv. Manag..

[209] Babcock-Jackson L., Carmichael W.W., Culver D.A. (2002). Dreissenid mussels increase exposure of benthic and pelagic organisms to toxic microcystins. Int. Ver. Theor. Angew. Limnol..

[210] Boegehold A.G., Johnson N.S., Kashiana D.R. (2019). Dreissenid (quagga and zebra mussel) veligers are adversely affected by bloom forming cyanobacteria. Ecotoxicol. Environ. Saf..

[211] Burmester V., Nimptsch J., Wiegand C. (2012). Adaptation of freshwater mussels to cyanobacterial toxins: Response of the biotransformation and antioxidant enzymes. Ecotoxicol. Environ. Saf..

[212] Juhel G., Davenport J., O’Halloran J., Culloty S.C., Ramsay R., James K.F., Furey A., Allis O. (2006). Pseudodiarrhoea in zebra mussels *Dreissena polymorpha* (Pallas) exposed to microcystins. J. Exp. Biol..

[213] Juhel G., O’Halloran J., Culloty S.C., O’riordan R.M., Davenport J., O’Brien N.M., James K.F., Furey A., Allis O. (2007). In vivo exposure to microcystins induces DNA damage in the haemocytes of the zebra mussel, *Dreissena polymorpha*, as measured with the comet assay. Environ. Mol. Mutagen..

[214] Juhel G., Ramsay R.M., Davenport J., O’Halloran J., Culloty S.C. (2015). Effect of the Microcystin-Producing Cyanobacterium, *Microcystis aeruginosa*, on Immune Functions of the Zebra Mussel *Dreissena polymorpha*. J. Shellfish Res..

[215] Makhutova O.N., Protasov A.A., Gladyshev M.I., Sylaieva A.A., Sushchik N.N., Morozovskaya I.A., Kalachova G.S. (2013). Feeding spectra of bivalve mollusks *Unio* and *Dreissena* from Kanevskoe Reservoir, Ukraine: Are they food competitors or not?. Zool. Stud..

[216] Paldavičienė A., Zaiko A., Mazur-Marzec H., Razinkovas-Baziukas A. (2015). Bioaccumulation of microcystins in invasive bivalves: A case study from the boreal lagoon ecosystem. Oceanologia.

[217] Pires L.M., Karlsson K.M., Meriluoto J.A., Kardinaal E., Visser P.M., Siewertsen K., Donk E.V., Ibelings B.W. (2004). Assimilation and depuration of microcystin-LR by the zebra mussel, *Dreissena polymorpha*. Aquat. Toxicol..

[218] Poste A.E., Ozersky T. (2013). Invasive dreissenid mussels and round gobies: A benthic pathway for the trophic transfer of microcystin. Environ. Toxicol. Chem..

[219] Sipiä V.O., Kankaanpää H.T., Pflugmacher S., Flinkman J., Furey A., James K.J. (2002). Bioaccumulation and detoxication of nodularin in tissues of flounder (*Platichthys flesus*), mussels (*Mytilus edulis, Dreissena polymorpha*), and clams (*Macoma balthica*) from the northern Baltic Sea. Ecotoxicol. Environ. Saf..

[220] Boltovskoy D., Correa N., Bordet F., Leites V., Cataldo D. (2013). Toxic *Microcystis* (cyanobacteria) inhibit recruitment of the bloom-enhancing invasive bivalve *Limnoperna fortunei*. Freshw. Biol..

[221] Gérard C., Poullain V., Lance E., Acou A., Brient L., Carpentier A. (2009). Influence of toxic cyanobacteria on community structure and microcystin accumulation of freshwater molluscs. Environ. Pollut..

[222] Saker M.L., Metcalf J.S., Codd G.A., Vasconcelos V.M. (2004). Accumulation and depuration of the cyanobacterial toxin cylindrospermopsin in the freshwater mussel *Anodonta cygnea*. Toxicon.

[223] Pereira P., Dias E., Franca S., Pereira E., Carolino M., Vasconcelos V. (2004). Accumulation and depuration of cyanobacterial paralytic shellfish toxins by the freshwater mussel *Anodonta cygnea*. Aquat. Toxicol..

[224] Eriksson J.E., Meriluoto J.A.O., Lindholm T. (1989). Accumulation of a peptide toxin from the cyanobacterium *Oscillatoria agardhii* in the freshwater mussel *Anadonta cygnea*. Hydrobiologia.

[225] Lindholm T., Eriksson J.E., Meriluoto J.A.O. (1989). Toxic cyanobacteria and water quality problems-Examples from a eutrophic lake on Åland, South West Finland. Water Res..

[226] Prepas E.E., Kotak B.G., Campbell L.M., Evans J.C., Hrudey S.E., Holmes C.F.B. (1997). Accumulation and elimination of cyanobacterial hepatotoxins by the freshwater clam *Anodonta grandis simpsoniana*. Can. J. Fish. Aquat. Sci..

[227] Chen J., Xie P., Guo L., Zheng L., Ni L. (2005). Tissue distributions and seasonal dynamics of the hepatotoxic microcystins-LR and -RR in a freshwater snail (*Bellamya aeruginosa*) from a large shallow, eutrophic lake of the subtropical China. Environ. Pollut..

[228] Watanabe M.F., Park H.D., Kondo F., Harada K., Hayashi H., Okino T. (1997). Identification and estimation of microcystins in freshwater mussels. Nat. Toxins.

[229] Yokoyama A., Park H.D. (2002). Mechanism and prediction for contamination of freshwater bivalves (Unionidae) with the cyanobacterial toxin microcystin in hypereutrophic Lake Suwa, Japan. Environ. Toxicol..

[230] Wu J.L., Liu W.X., Wen C.G., Qian G.M., Hu B.Q., Jian S.Q., Yang G., Dong J. (2020). Effect of microcystin on the expression of Nrf2 and its downstream antioxidant genes from *Cristaria plicata*. Aquat. Toxicol..

[231] Wu J., Liu W., Hou S., Wang Y., Fang H., Luo S., Yang L., Wen C. (2023). Identification of Nrf2/Keap1 pathway and its transcriptional regulation of antioxidant genes after exposure to microcystins in freshwater mussel *Cristaria plicata*. Dev. Comp. Immunol..

[232] Travers B., Murby A., Haney J.F. (2011). Bioaccumulation of Microcystins by Freshwater Mussels in Mystic Lake and Middle Pond, MA. UNH Cent. Freshw. Biol. Res..

[233] Chen J., Xie P. (2005). Seasonal dynamics of the hepatotoxic microcystins in various organs of four freshwater bivalves from the large eutrophic lake Taihu of subtropical China and the risk to human consumption. Environ. Toxicol..

[234] Yokoyama A., Park H.D. (2003). Depuration kinetics and persistence of the cyanobacterial toxin microcystin-LR in the freshwater bivalve *Unio douglasiae*. Environ. Toxicol..

[235] Lance E., Brient L., Carpentier A., Acou A., Marion L., Bormans M., Gérard C. (2010). Impact of toxic cyanobacteria on gastropods and microcystin accumulation in a eutrophic lake (Grand-Lieu, France) with special reference to *Physa* (=*Physella*) *acuta*. Sci. Total Environ..

[236] Kotak B.G., Zurawell R.W., Prepas E.E., Holmes C.F.B. (1996). Microcystin-LR concentration in aquatic food web compartments from lakes of varying trophic status. Can. J. Fish. Aquat. Sci..

[237] Zurawell R.W., Kotak B.G., Prepas E.E. (1999). Influence of lake trophic status on the occurrence of microcystin-LR in the tissue of pulmonate snails. Freshw. Biol..

[238] Lance E., Brient L., Bormans M., Gérard C. (2006). Interactions between cyanobacteria and gastropods I. Ingestion of toxic *Planktothrix agardhii* by *Lymnaea stagnalis* and the kinetics of microcystin bioaccumulation and detoxification. Aquat. Toxicol..

[239] Lance E., Paty C., Bormans M., Brient L., Gérard C. (2007). Interactions between cyanobacteria and gastropods II. Impact of toxic *Planktothrix agardhii* on the life-history traits of *Lymnaea stagnalis*. Aquat. Toxicol..

[240] Lance E., Josso C., Dietrich D., Ernst B., Paty C., Senger F., Bormans M., Gérard C. (2010). Histopathology and microcystin distribution in *Lymnaea stagnalis* (Gastropoda) following toxic cyanobacterial or dissolved microcystin-LR exposure. Aquat. Toxicol..

[241] Lance E., Neffling M.R., Gérard C., Meriluoto J., Bormans M. (2010). Accumulation of free and covalently bound microcystins in tissues of *Lymnaea stagnalis* (Gastropoda) following toxic cyanobacteria or dissolved microcystin-LR exposure. Environ. Pollut..

[242] Lance E., Alonzo F., Tanguy M., Gérard C., Bormans M. (2011). Impact of microcystin-producing cyanobacteria on reproductive success of *Lymnaea stagnalis* (Gastropoda, Pulmonata) and predicted consequences at the population level. Ecotoxicology.

[243] Lance E., Petit A., Sanchez W., Paty C., Gérard C., Bormans M. (2014). Evidence of trophic transfer of microcystins from the gastropod *Lymnaea stagnalis* to the fish *Gasterosteus aculeatus*. Harmful Algae.

[244] Lance E., Desprat J., Holbech B.F., Gérard C., Bormans M., Lawton L.A., Edwards C., Wiegand C. (2016). Accumulation and detoxication responses of the gastropod *Lymnaea stagnalis* to single and combined exposures to natural (cyanobacteria) and anthropogenic (the herbicide RoundUp^®^ Flash) stressors. Aquat. Toxicol..

[245] Zurawell R.W., Holmes C.F., Prepas E.E. (2006). Elimination of the cyanobacterial hepatotoxin microcystin from the freshwater pulmonate snail *Lymnaea stagnalis jugularis* (say). J. Toxicol. Environ. Health A.

[246] Zurawell R.W., Goldberg J.I., Holmes C.F., Prepas E.E. (2007). Tissue distribution and oral dose effects of microcystin in the freshwater pulmonate snail *Lymnaea stagnalis jugularis* (Say). J. Toxicol. Environ. Health A.

[247] Zhang J., Wang Z., Song Z., Xie Z., Li L., Song L. (2012). Bioaccumulation of microcystins in two freshwater gastropods from a cyanobacteria-bloom plateau lake, Lake Dianchi. Environ. Pollut..

[248] Sitnikova T., Kiyashko S.I., Maximova N., Pomazkina G.V., Roepstorf P., Wada E., Michel E. (2012). Resource partitioning in endemic species of Baikal gastropods indicated by gut contents, stable isotopes and radular morphology. Hydrobiologia.

[249] He Q., Kang L., Sun X., Jia R., Zhang Y., Ma J., Li H., Ai H. (2018). Spatiotemporal distribution and potential risk assessment of microcystins in the Yulin River, a tributary of the Three Gorges Reservoir, China. J. Hazard. Mater..

[250] Lance E., Bugajny E., Bormans M., Gerard C. (2008). Consumption of toxic cyanobacteria by *Potamopyrgus antipodarum* (Gastropoda, Prosobranchia) and consequences on life traits and microcystin accumulation. Harmful Algae.

[251] White S.H., Duivenvoorden L.J., Fabbro L.D., Eaglesham G.K. (2006). Influence of intracellular toxin concentrations on cylindrospermopsin bioaccumulation in a freshwater gastropod (*Melanoides tuberculata*). Toxicon.

[252] Qiao F., Lei K., Han X., Wei Z., Zhao X., An L., LeBlanc G.A. (2018). No impacts of microcystins on wild freshwater snail *Bellamya Aeruginosa* fecundity from a eutrophic lake. Environ. Toxicol. Pharmacol..

[253] Zhang D., Xie P., Liu Y., Chen J., Liang G. (2007). Bioaccumulation of the hepatotoxic microcystins in various organs of a freshwater snail from a subtropical Chinese lake, Taihu Lake, with dense toxic Microcystis blooms. Environ. Toxicol. Chem..

[254] Zhang D., Xie P., Liu Y., Chen J., Wen Z. (2009). Spatial and temporal variations of microcystins in hepatopancreas of a freshwater snail from Lake Taihu. Ecotoxicol. Environ. Saf..

[255] Xie L., Yokoyama A., Nakamura K., Park H. (2007). Accumulation of microcystins in various organs of the freshwater snail *Sinotaia histrica* and three fishes in a temperate lake, the eutrophic Lake Suwa, Japan. Toxicon.

[256] Xie L., Hanyu T., Futatsugi N., Komatsu M., Steinman A.D., Park H.D. (2014). Inhibitory effect of naringin on microcystin-LR uptake in the freshwater snail *Sinotaia histrica*. Environ. Toxicol. Pharmacol..

[257] Czyżewska W., Piontek M., Łuszczyńska K. (2020). The Occurrence of Potential Harmful Cyanobacteria and Cyanotoxins in the Obrzyca River (Poland), a Source of Drinking Water. Toxins.

[258] White S., Duivenvoorden L., Fabbro L. (2005). Impacts of a Toxic *Microcystis* Bloom on the Macroinvertebrate Fauna of Lake Elphinstone, Central Queensland, Australia. Hydrobiologia.

[259] Caro Borrero A., Carmona Jiménez J., Márquez Santamaría K., Elvira P. (2021). Relationships between environmental conditions and macroalgae structure on the benthic macroinvertebrate establishment: Diversity and conservation in rivers of central Spain and Mexico. J. Insect Conserv..

