# Supplementary material for: Cyanobacteria and Macroinvertebrate Relationships in Freshwater Benthic Communities beyond Cytotoxicity
Source: Toxins (Basel). 2024 Apr 15;16(4):190. doi: 10.3390/toxins16040190 (PMC11054157; doi:10.3390/toxins16040190)
Supplement: Supplementary file 1 [file toxins-16-00190-s001.zip › toxins-2919018-supplementary.pdf]

Table S1 (Suppl. Mat.). Compilation of data regarding the relationships between cyanobacteria and benthic macroinvertebrates. Species and associated data are grouped in families, which is the most used taxonomic level in scientific studies. Parameters influencing the relationships between macroinvertebrates and cyanobacteria have been organized into columns, including the life cycle stage (if known, the substage or size of larval or juvenile stage is indicated), the origin of the macroinvertebrate specimens analyzed (lakes, rivers, etc.), the type of assay conducted (field observations or laboratory trials), the cyanobacteria associated with and their toxicity if explicitly indicated in the study (denoted with the signs + and -), the type of cyanotoxin used, aspects of the relationships studied simplified into topics and bibliographic references where the indicated information can be found (**A**, adult; **ANTX**, anatoxins; **BMAA**, β-N-methylamino-L-alanine; **CB**, cell-bound toxins; **CF**, cell-free toxins; **Co**, Cohabiting; **CYN**, Cylindrospermopsin; **EX**, extracts; **F**, feeding; **J**, juvenile; **L**, larva; **Lab**, laboratory assays; **MC**, microcystins; **Nat**, nature observations; **NOD**, nodularins; **NS**, not studied; **P**, pupa; **PST**, paralytic shellfish toxins; **U**, unknow).

Macroinvertebrate taxa	Instar	Habitat	Data	Cyanobacteria	Toxins	Exposure	Research topics	References
**Arthropoda, Hexapoda, Diptera, Chaoboridae**								
Chaoborus sp.	L (4)	Unknow	Lab	*Microcystis aeruginosa* (+)	NS	F, CB	Survival/Mortality test; forced feeding on prey toxins accumulated; Toxins food web transfer	[138]
**Arthropoda, Hexapoda, Diptera, Chironomidae**								
Chaetocladius sp.	L	Streams	Nat	*Chamaesiphon curvatus, C. polonicus*, *Phormidium* sp.	NS	F, Co	Wild feeding on cyanobacteria; Gut content analysis; Tolerance to habitats abundant in cyanobacteria	[139]
Chironomidae	L	Water bodies	Nat	*Oscillatoria* sp.; Cyanobacteria sediments; Cyanobacteria blooms (*Microcystis* sp.); Metaphyton (*Lyngbya Wollei*)	NS; MCs	F, Co	Wild feeding on cyanobacteria; Gut contents and food selection analysis; colonies, mats or mass of cyanobacteria; Paleoecological sediment studies: cyanotoxins conditioned chironomids community; Food assimilation analysis (cyanobacteria cells survival to intestinal transit); Cyanobacteria bloom; Abundance of macroinvertebrates and cyanobacteria; Cyanobacteria determine the distribution and abundance of midge populations	[139,140,141,142,143,144,145]
Chironomus spp.	L	Lakes; Rivers	Lab+Nat	*Planktothrix agardhii, Dolichospermum* spp., *Cuspidothrix issatschenkoi*; *Anabaena* sp.	MC-LA, -LR -RR, -YR, ANTX-a extracts	F, CF	Wild feeding on cyanobacteria; Gut contents analysis; Toxins bioaccumulation; Survival/mortality; Tolerance habitats abundant in cyanobacteria; Effects of single toxins, mixture, or extracts	[90,124]
Chironomus balatonicus Dévai, Wülker & Scholl, 1983	L	Streams	Nat	Anabaena spp., *Chroococcales* sp., *Microcystis* spp., *Oscillatoria* spp., *Woronichinia compacta*	NS	F	Wild feeding on cyanobacteria; Gut content analysis; Food assimilation analysis (midge mouthpart and algal morphology)	[146]

Chironomus crassicaudatus Malloch, 1915	L (4)	Lakes	Lab	*Anabaena* sp., *Anabaena flos-aquae*, *Lyngbya* sp., *Lyngbya aeruginosa*, *Microcystis* sp., *Anacystis* sp., *Gloeocapsa* sp.	NS	F	Forced feeding on cyanobacteria; Food selection analysis; Digestibility; Territorial distribution conditioned by cyanobacteria	[88,147,148]
Chironomus decorus Johansen, 1905	L	Lakes	Nat	Not identified	NS	F	Wild feeding on cyanobacteria; Gut contents analysis; Territorial distribution conditioned by cyanobacteria	[148,149]
Chironomus dilutus Shobanov, Kiknadze & Butler, 1999	L	Water bodies	Lab	*Phormidium* sp (strain 1 (+): *P.* cf. *subfuscum*; strain 2 (+): *P.* cf. *irriguum;* strain 3 (+): *P.* cf. *autumnale*)	ANTX	CF	Survival/mortality; Harmful effect of cyanobacteria extracts	[150]
Chironomus pallidivittatus Malloch, 1915	L (1-4)	Lakes	Lab	*Microcystis aeruginosa (+); M. wesenbergii (-)*	MC-LR	F, CF	Survival/mortality; Harmful effect of single toxins, mixture or extracts on behavior, immature growth, cell and tissue and metabolism; Metabolic detoxification processes (oxidative stress)	[31,151]
Chironomus plumosus (Linnaeus, 1758)	L	Streams	Nat	*Aphanizomenon flos-aquae*, Chroococcales spp., *Microcystis* spp., *Oscillatoria* spp., *Snowella lacustris, Woronichinia compacta*	NS	F	Wild feeding on cyanobacteria; Gut content analysis; Food assimilation analysis (midge mouthpart and algal morphology)	[146]
Chironomus riparius Meigen, 1804	L	Stock culture	Lab	*Anabaena* sp., *Spirulina* sp. *Trichormus variabilis* (+)	MC-LR; extracts	F, CF	Forced feeding on cyanobacteria; Food assimilation analysis (isotope labeling); Life cycle conclusion; Survival/mortality; Toxicity synergies; Harmful effect on immature growth, life cycle, molecular, tissue and metabolism; Metabolic detoxification processes (oxidative stress: CAT, GST, GSH and LPO)	[100,102,152,153,154]
Cladotanytarsus mancus (Walker, 1856)	L	Streams; Lakes	Nat	*Chroococcus* sp, *Kamptonema formosum*, *Leptolyngbya boryana* and *Oscillatoria princeps*	NS	F	Gut contents analysis	[92]
Conchapelopia melanops (Meigen, 1818)	L (4)	Rivers	Nat	Not identified	NS	Co	Metabolic detoxification processes (oxidative stress: CAT, GST, GSH and LPO)	[154]
Cricotopus sp.	L	Streams	Nat	*Nostoc parmelioides; N. verrucosum*	NS	F, Co	Macroinvertebrate-cyanobacteria mutualism; Inhabit exclusively cyanobacteria; Wild feeding on cyanobacteria	[96,117]
Cricotopus cataractaenostocicola (Tachibana, 2022)	L	Streams	Nat	*Nostoc* sp.	NS	F, Co	Macroinvertebrate-cyanobacteria mutualism; Inhabit exclusively cyanobacteria; Wild feeding on cyanobacteria	[114]
Cricotopus fuscatus Wirth, 1957	L	Streams	Nat	*Nostoc parmelioides*	NS	F, Co	Macroinvertebrate-cyanobacteria mutualism; Inhabit exclusively cyanobacteria; Wild feeding on cyanobacteria	[93,155]
Cricotopus levantinus Moubayed & Hirvenoja, 1986	L	Streams	Nat	*Nostoc parmelioides*	NS	F, Co	Macroinvertebrate-cyanobacteria mutualism; Inhabit exclusively cyanobacteria; Wild feeding on cyanobacteria	[116]

Cricotopus lygropis Edwards 1929	L	Streams	Nat	*Nostoc parmelioides*	NS	F, Co	Macroinvertebrate-cyanobacteria mutualism; Inhabit exclusively cyanobacteria; Wild feeding on cyanobacteria; Gut contents analysis	[85]
Cricotopus nostocicola Wirth, 1957	L	Streams	Nat	*Nostoc parmelioides*	NS	F, Co	Macroinvertebrate-cyanobacteria mutualism; Inhabit exclusively cyanobacteria; Wild feeding on cyanobacteria	[93,115,155,156]
Cricotopus sylvestris (Fabricius, 1794)	L	Lakes		*Microcistis* sp., *Oscillatoria* sp., *Planktothrix* sp.	NS	F	Wild feeding on cyanobacteria; Gut contents analysis	[157]
Cryptochironomus defectus (Kieffer, 1913)	L	Streams; Lakes	Nat	*Chroococcus* sp, *Kamptonema formosum*, *Leptolyngbya boryana* and *Oscillatoria princeps*	NS	F	Gut contents analysis	[92]
Cryptochironomus fulvus Johannsen, 1905	L	Lakes	Nat	Cyanobacteria	NS	Co	Tolerance habitats abundant in cyanobacteria; Territorial distribution conditioned by cyanobacteria	[148]
Diamesa sp.	L	Streams	Nat	*Chamaesiphon curvatus, C.*, *Phormidium* sp.	NS	F, Co	Wild feeding on cyanobacteria; Gut content analysis; Tolerance to habitats abundant in cyanobacteria	[139]
Diamesa latitarsis (Goetghebuer, 1921)	L	Streams	Nat	*Chamaesiphon curvatus, C.*, *Phormidium* sp.	NS	F, Co	Wild feeding on cyanobacteria; Gut content analysis; Tolerance to habitats abundant in cyanobacteria	[139]
Diamesa nowickiana Kownacki, 1975	L	Streams	Nat	*Chamaesiphon curvatus, C.*, *Phormidium* sp.	NS	F, Co	Wild feeding on cyanobacteria; Gut content analysis; Tolerance to habitats abundant in cyanobacteria	[139]
Diamesa steinboecki Goetghebuer, 1933	L	Streams	Nat	*Chamaesiphon curvatus, C.*, *Phormidium* sp.	NS	F, Co	Wild feeding on cyanobacteria; Gut content analysis; Tolerance to habitats abundant in cyanobacteria	[139]
Diamesa ursus (Kieffer, 1918)	L	Streams	Nat	*Chamaesiphon curvatus, C.*, *Phormidium* sp.	NS	F, Co	Wild feeding on cyanobacteria; Gut content analysis; Tolerance to habitats abundant in cyanobacteria	[139]
Glyptotendipes sp	L	Lakes		*Oscillatoria* sp., *Planktothrix* sp.	NS	F	Wild feeding on cyanobacteria; Gut contents analysis	[157]
Glyptotendipes paripes (Edwards, 1929)	L	Lakes	Lab	Cyanobacteria, *Anabaena flos-aquae*, *Lyngbya aeruginosa*, *Microcystis* sp.	NS	F, Co	Forced feeding on cyanobacteria; Food selection, gut contents sand food assimilation analysis (algal morphology); Digestibility; Life cycle conclusion; Harmful effect on immature growth; Territorial distribution conditioned by cyanobacteria	[89,148,149]
Glyptotendipes tokunagai Sasa, 1979	L	Stock culture	Lab	*Microcystis aeruginosa (+); M. wesenbergii (-)*	MC-LR	Co	Bio flocculation (large algal aggregates); Natural water detoxification; Midge silk	[158]
Endochironomus albipennis (Meigen, 1830)	L	Lakes		*Limnothrix* sp., *Microcystis* sp., *Oscillatoria* sp., *Planktothrix* sp., *Anabaena* sp.	NS	F	Wild feeding on cyanobacteria; Gut contents analysis	[157]
Labrundinia sp	L	Rivers	Nat	*Anabaena* sp.	NS	F	Wild feeding on cyanobacteria; Gut contents analysis	[159]
Micropsectra sp.	L	Streams	Nat	*Nostoc parmelioides*	NS	Co	Tolerance to habitats abundant in cyanobacteria	[112]
Omisus sp.	L	Rivers	Nat	*Anabaena* sp.	NS	F	Wild feeding on cyanobacteria; Gut contents analysis	[159]
Orthocladius rivicola Kieffer, 1911	L	Streams	Nat	*Chamaesiphon curvatus, C. polonicus*, *Phormidium* sp.	NS	F, Co	Wild feeding on cyanobacteria; Gut content analysis; Tolerance to habitats abundant in cyanobacteria	[139]
Orthocladius thienemanni Kieffer & Thienemann, 1906	L	Streams	Nat	*Chamaesiphon curvatus, C. polonicus*, *Phormidium* sp.; *Tolypothrix tenuis*, *Calothrix*	NS	F, Co	Wild feeding on cyanobacteria; Gut content analysis; Tolerance to habitats abundant in cyanobacteria	[139,160]

				gypsophila, *Coelosphaerium kuetzingianum*, *Gomphosphaeria lacustris*				
	Pagastia partica (Roback, 1957)	L	Rivers	Nat	*Nostoc* sp.	NS	F, Co	Wild feeding on cyanobacteria; Gut content analysis; Tolerance to habitats abundant in cyanobacteria	[161]
	Parakiefferiella sp.	L	Rivers	Nat	*Anabaena* sp.	NS	F	Wild feeding on cyanobacteria; Gut contents analysis	[159]
	Parametriocnemus sp.	L	Rivers	Nat	*Anabaena* sp.	NS	F	Wild feeding on cyanobacteria; Gut contents analysis	[159]
	Paratanytarsus sp.	L (4)	Rivers	Nat	Not identified	NS	Co	Metabolic detoxification processes (oxidative stress: CAT, GST, GSH and LPO)	[154]
	Polypedilum scalaenum (Schrank, 1803)	L	Streams; Lakes	Nat	*Chroococcus* sp, *Kamptonema formosum*, *Leptolyngbya boryana* and *Oscillatoria princeps*	NS	F	Gut contents analysis	[92]
	Polypedilum sp.	L	Rivers	Nat	*Anabaena* sp.	NS	F	Wild feeding on cyanobacteria; Gut contents analysis	[159]
	Prodiamesa olivacea (Meigen, 1818)	L (4)	Rivers	Nat	Not identified	NS	Co	Metabolic detoxification processes (oxidative stress: CAT, GST, GSH and LPO)	[154]
	Pseudodiamesa nivosa Goetgehebuer, 1928	L	Streams	Nat	*Chamaesiphon curvatus, C. polonicus*, *Phormidium* sp.	NS	F, Co	Wild feeding on cyanobacteria; Tolerance to habitats abundant in cyanobacteria	[139]
	Pseudokiefferiella parva (Edwards, 1932)	L	Streams	Nat	*Chamaesiphon curvatus, C. polonicus*, *Phormidium* sp.	NS	F, Co	Wild feeding on cyanobacteria; Tolerance to habitats abundant in cyanobacteria	[139]
	Tanypodinae	L	Lakes	Nat	Cyanobacteria	NS	Co	Tolerance habitats abundant in cyanobacteria; Territorial distribution conditioned by cyanobacteria	[148]
	Tanytarsus sp.	L	Rivers	Nat	*Anabaena* sp.	NS	F	Wild feeding on cyanobacteria; Gut contents analysis	[159]
	Tanypus chinensis Wang, 1994	L	Lakes	Nat	*Microcystis* sp., *Oscillatoria* sp., *Dolichospermum* sp.	MC-LR, -RR, -YR	F, CF	Toxin bioaccumulation	[97]
	Tendipes decorus (Johannsen, 1905)	L	Lakes	Nat	*Microcystis* sp.	NS	F	Wild feeding on cyanobacteria; Gut contents analysis	[149]
**Arthropoda, Hexapoda, Diptera, Ceratopogonidae**									
	Forcipomyia spp.	L	Unknow	Lab	*Anabaena* sp.	NS	F	Forced feeding on cyanobacteria; Life cycle conclusion	[162]
	Forcipomyia intonsa Chan and LeRoux, 1971	P	Reservoir	Nat	Cyanobacteria	NS	Co	Tolerance to habitats abundant in cyanobacteria	[163]
	Forcipomyia taiwana (Shiraki, 1913)	L	Lakes	Lab	*Anabaena* sp.	NS	F	Forced feeding on cyanobacteria; Life cycle	[162]
**Arthropoda, Hexapoda, Diptera, Ephydridae**									
	Axysta cesta (Haliday, 1833)	L	water bodies shores and wetland	Nat	*Lyngbya* sp., *Cylindrospermun*	NS	F	Wild and forced feeding on cyanobacteria; Trophic specialist	[164]
	Coenia curvicauda (Meigen, 1830)	L; A	water bodies shores and wetland	Lab+Nat	*Anabaena flos-aquae*, *A. variabilis*, *Calothrix* sp., *Cylindrospermum* sp., *Gloeocapsa alpicola*, *Lyngbya spiralis*, *Oscillatoria tenuis*, *Synechococcus leopoliensis*	NS	F	Wild and forced feeding on cyanobacteria; Gut content analysis; Life cycle	[165,166]

Dichaeta caudata (Fallén, 1813)	L	water bodies shores and wetland	Nat	Filamentous cyanobacteria	NS	Co	Tolerance habitats abundant in cyanobacteria	[166]
Discocerina obscurella (Fallén, 1813)	L	Water bodies shores and wetland	Nat	Unicellular and filamentous cyanobacteria	NS	Co	Tolerance habitats abundant in cyanobacteria	[166]
Ephydra bruesi (Cresson, 1934)	L	Spring	Lab+Nat	Cyanobacteria mats	NS	F	Food assimilation analysis (isotope labeling)	[167]
Ephydra cinerea (Jones, 1906)	L	Lakes	Nat	Gelatinous blue-green algal masses.	NS	Co	Territorial distribution conditioned by cyanobacteria	[168]
Ephydra macellaria Egger, 1862	L; A	Lakes	Nat	Cyanobacteria mats	NS	F, C o	Wild feeding on cyanobacteria; Inhabit colonies or mats of cyanobacteria	[118]
Ephydra riparia Fallén, 1813	L	Water bodies shores and wetland	Lab	*Gloeocapsa* sp., *Anabaena* sp.	NS	F	Forced feeding on cyanobacteria; Life cycle	[169]
Ephydra termophila Cresson, 1934	L	Alkaline hot spring	Lab	*Phormidium* sp., *Mastigocladus* sp., *Oscillatoria* sp.	NS	F	Forced feeding on cyanobacteria; Life cycle	[170]
Hyadina albovenosa Coquillett, 1900	L; A	Wetland	Lab+Nat	*Anabaena flos-aquae*, *A. variabilis*, *Cylindrospermum* sp., *Gloeocapsa alpicola*, *Lyngbya spiralis*, *Nostoc commune*, *Oscillatoria tenuis*, *Spirulina* sp., *Symploca muscorum*, *Synechococcus leopoliensis*	NS	F	Food selection analysis; forced feeding on cyanobacteria; Life cycle	[86]
Hyadina binotata (Cresson, 1926)	L	Wetland	Nat	*Anabaena* sp., *Cylindrospermum* sp., *Lyngbya* sp., *Phormidium* sp.	NS	F	Trophic specialist; Inhabit colonies or mats of cyanobacteria; Wild feeding study on cyanobacteria	[83,164]
Hyadina neglecta Sturtevant & Wheeler, 1954	L	Wetland	Nat	*Cylindrospermum* sp	NS	F	Trophic specialist; Wild feeding study on cyanobacteria	[164]
Hyadina subnitida Sturtevant & Wheeler 1954	L	Wetland	Nat	*Anabaena* sp., *Anacystis* sp., *Cylindrospermum* sp., *Lyngbya* sp., *Phormidium* sp.	NS	F	Trophic specialist; Inhabit colonies or mats of cyanobacteria; Wild feeding study on cyanobacteria	[83,164]
Hydrellia formosa Loew, 1861	L	Water bodies shores	Nat	*Nostoc* sp., *Aphanocapsa* sp.	NS	Co	Inhabit colonies, mats or mass of cyanobacteria	[166]
Hydrellia harti Cresson, 1936	L	Water bodies shores	Nat	*Nostoc* sp., *Aphanocapsa* sp.	NS	Co	Inhabit colonies, mats or mass of cyanobacteria	[166]
Lytogaster abdominalis (Stenhammar, 1844)	L	Water bodies shores and wetland	Nat	*Cylindrospermum* sp.	NS	F	Trophic specialist; Wild feeding study on cyanobacteria	[164]
Lytogaster excavata (Sturtevant & Wheeler, 1954)	L	Water bodies shores and wetland	Lab+Nat	*Anabaena variabilis*; *A. fios-aquae*, *Anabaena* sp., *Anacystis nidulans*, *Cylindrospermum* sp, *Gloecapsa alpicola*, *Lyngbya* sp., *Nostoc*	NS	F	Trophic specialist; Wild and forced feeding on cyanobacteria; Life cycle	[83,164,171]

				commune, *Oscillatoria chalybea*, *Oscillatoria tenuis*, *Phormidium* sp., *Synechococcus leopoliensis*				
Lytogaster flavipes (Sturtevant & Wheeler, 1954)	L	Water bodies shores and wetland	Nat	*Cylindrospermum* sp	NS	F	Trophic specialist; Wild feeding on cyanobacteria	[164]
Lytogaster furva (Cresson, 1926*)*	L	Water bodies shores and wetland	Lab+Nat	*Cylindrospermum* sp	NS	F	Trophic specialist; Inhabit colonies or mats of cyanobacteria; Wild feeding on cyanobacteria	[164]
Nostima approximata Sturtevant & Wheeler, 1954	L	Wetland	Lab+Nat	*Anabaena flos-aquae, A. variabilis; Anabaena sp.,* Cylindrospermum *sp, Gloeocapsa alpicola, Lyngbya spiralis., Nostoc commune, Oscillatoria limosa, O. tenuis, Phormidium sp., Scytonema sp., Symploca muscorum, Synechococcus leopoliensis*	NS	F	Trophic specialist; Wild and forced feeding on cyanobacteria; Life cycle	[140]
Paracoenia turbida (Curran, 1927)	L; A	Alkaline hot spring	Lab+Nat	Cyanobacteria mats formed mainly by *Phormidium* sp., *Mastigocladus sp.* and *Oscillatoria* sp.	NS	F, Co	Wild and forced feeding on cyanobacteria; Gut contents and food assimilation (isotope labeling) analysis; Inhabit colonies, mats or mass of cyanobacteria	[167,172,173]
Parydra aquila (Fallen, 1813)	L	Water bodies shores and wetland	Nat	*Oscillatoria* sp.	NS	F	Wild feeding on cyanobacteria; Gut contents analysis	[164, 166, 169]
Parydra quadrituberculata (Loew, 1862)	L	Water bodies shores and wetland	Nat	*Oscillatoria* sp.	NS	F	Wild feeding on cyanobacteria; Gut contents analysis	[164,166,169]
Pelina truncatula (Loew, 1878)	L	Water bodies shores and wetland	Nat	*Anabaena variabilis*; *A. fios-aquae*, *Anabaena* sp., *Cylindrospermum* sp, *Gloecapsa alpicola*, *Gloecapsa* sp., *Lyngbya spiralis*., *Nostoc commune*, *Oscillatoria sp.*, *O. tenuis*, *Phormidium* sp., *Synechococcus leopoliensis*	NS	F	Trophic specialist; Wild and forced feeding on cyanobacteria; Life cycle	[164,174]
Scatella picea (Walker, 1849)	L	Water bodies shores and wetland	Nat	*Anabaena flos-aquae*, *A. variabilis*, *Cylindrospermum* sp., *Gloeocapsa* sp., *Lyngbya* sp., *Nostoc commune*	NS	F	Forced feeding on cyanobacteria; Life cycle	[175]
Scatella stagnalis Fallén, 1813	L; A	Water bodies shores and wetland	Lab+Nat	*Nostoc* sp., *Nostoc muscorum*, *N. commune*, *Gloeocapsa* sp., *Anabaena* sp., *Anabaena flos-aquae*, *Cylindrospermum* sp.	NS	F	Trophic specialist; Wild and forced feeding on cyanobacteria; Life cycle	[164,176]

Setacera atrovirens (Loew, 1862)	L	Water bodies shores and wetland	Lab+Nat	*Anabaena variabilis; Anabaena sp., Anacystis sp., Cylindrospermum sp, Gloeocapsa sp., Lyngbya sp., Nostoc sp., Nostoc commune, Oscillatoria sp., Oscillatoria chalybea, O. tenuis, Phormidium sp., Synechococcus leopoliensis*	NS	F	Trophic specialist; Wild and forced feeding on cyanobacteria; Life cycle	[164,169,177]
Setacera pacifica (Cresson, 1925)	L	Highly alkaline ponds	Lab+Nat	*Anabaena* sp., *Oscillatoria* sp.; *Nostoc* sp.	NS	F	Trophic specialist; Wild and forced feeding on cyanobacteria; Life cycle	[164,177]
Setacera durani (Cresson, 1935)	L	Streams	Nat	Cyanobacteria mats	NS	Co	Inhabit colonies, mats or mass of cyanobacteria	[177]
**Arthropoda, Hexapoda, Diptera, Tipulidae**								
Dicranota bimaculata (Schummel, 1829)	L	Rivers	Nat	*Tolypothrix tenuis, Calothrix gypsophila, Coelosphaerium kuetzingianum, Gomphosphaeria lacustris*	NS	F	Wild feeding on cyanobacteria; Gut contents analysis	[160]
**Arthropoda, Hexapoda, Ephemeroptera, Ameletiidae**								
Ameletus similior McDunnough, 1928	L	Streams	Lab	*Nostoc parmelioides*	NS	Co	Tolerance habitats abundant in cyanobacteria	[112]
**Arthropoda, Hexapoda, Ephemeroptera, Baetidae**								
Baetiella pseudofrequenta (Müller-Liebenau, 1985)	L	Rivers	Nat	*Calothrix* sp., *Microcystis* sp., *Phormidium* sp.	NS	F	Wild feeding on cyanobacteria; Food assimilation analysis (isotope labeling)	[98]
Baetis bicaudatus Dodds, 1923	L	Streams	Lab	*Nostoc parmelioides*	NS	Co	Tolerance habitats abundant in cyanobacteria	[113]
Cloeon dipterum (Linnaeus, 1761)	5-6 mm	Reservoir	Lab+Nat	*Aphanizomenon* sp.	NS	F	Wild feeding on cyanobacteria; Food assimilation analysis (isotope labeling 13C and 15N);	[178]
Procloeon venustum	L	Rivers	Nat	*Calothrix* sp., *Microcystis* sp., *Phormidium* sp.	NS	F	Wild feeding on cyanobacteria; Food assimilation analysis (isotope labeling)	[98]
**Arthropoda, Hexapoda, Ephemeroptera, Caenidae**								
Caenis spp.	L	Rivers	Nat	*Calothrix* sp., *Microcystis* sp., *Phormidium* sp.	NS	F	Wild feeding on cyanobacteria; Food assimilation analysis (isotope labeling)	[98]
**Arthropoda, Hexapoda, Ephemeroptera, Ephemerellidae**								
Ephemerella sp.	L	Lakes	Lab	*Microcystis aeruginosa (+)*	NS	CB	Survival/mortality	[153]
Eurylophella sp.	L	Lakes	Lab	*Microcystis aeruginosa (+)*	NS	CB	Survival/mortality	[153]
Caurinella idahoensis (Allen, 1984)	L	Streams	Nat	*Nostoc parmelioides*	NS	Co	Tolerance habitats abundant in cyanobacteria; Inhabit colonies, mats or mass of cyanobacteria	[113]
**Arthropoda, Hexapoda, Ephemeroptera, Ephemeridae**								
Ephemera danica Müller, 1764	L	Streams	Nat	Cyanobacteria	NS	F	Gut contents analysis; Harmful effect on immature growth	[179]

Hexagenia spp.	L	Lakes, Rivers	Nat	Cyanobacteria blooms. *Microcystis aeruginosa*	MC-LR	CF	Toxin bioaccumulation; Survival/mortality; Harmful effect on reproduction, immature growth and life cycle; Toxicity synergies	[103,108,180]
Hexagenia limbata (Serville, 1829)	L, A	Lakes	Nat	*Microcystis aeruginosa (+)*	MC-LR, -LA, -RR, -YR	F, CF	Toxin bioaccumulation; food web toxin transfer	[106,134,181]
**Arthropoda, Hexapoda, Ephemeroptera, Heptageniidae**								
Afronurus spp	L	Rivers	Nat	*Calothrix* sp., *Microcystis* sp., *Phormidium* sp.	NS	F	Wild feeding on cyanobacteria; Food assimilation analysis (isotope labeling)	[98]
Cinygmina sp.	L	Rivers	Nat	*Calothrix* sp., *Microcystis* sp., *Phormidium* sp.	NS	F	Wild feeding on cyanobacteria; Food assimilation analysis (isotope labeling)	[98]
Cinygmula sp	L	Streams	Lab	*Nostoc parmelioides*	NS	Co	Tolerance habitats abundant in cyanobacteria	[113]
Ecdyonurus angelieri Thomas, 1968	L	River	Lab+Nat	Not identified	MC-LR, -LW	CF	Survival/mortality; Bioaccumulation; Harmful effect on cells or tissue;	[120]
**Arthropoda, Hexapoda, Ephemeroptera, Leptophlebiidae**								
Choroterpes spp.	L	Rivers	Nat	*Calothrix* sp., *Microcystis* sp., *Phormidium* sp.	NS	F	Wild feeding on cyanobacteria; Food assimilation analysis (isotope labeling)	[98]
Deleatidium spp.	L	Streams	Lab	*Microcoleus autumnalis*	ANTX-a, dihydroanatoxin-a and homoanatoxin-a / dihydrohomoanatoxin-a	Co	Survival/Mortality; Toxins bioaccumulation	[182]
Paraleptophlebia sp	L	Streams	Lab	*Nostoc parmelioides*	NS	Co	Tolerance habitats abundant in cyanobacteria	[113]
**Arthropoda, Hexapoda, Plecoptera, Capniidae**								
Allocapnia granulata (Claassen, 1924)	A	Rivers	Nat	Cyanobacteria	NS	F	Wild feeding on cyanobacteria	[183,184]
Allocapnia recta (Claassen, 1924)	A	Rivers	Nat	Cyanobacteria	NS	F	Wild feeding on cyanobacteria	[183,184]
Allocapnia vivipara (Claassen, 1924)	A	Rivers	Nat	Cyanobacteria	NS	F	Wild feeding on cyanobacteria	[183,184]
Allocapnia mystica Frison, 1929	A	Rivers	Nat	Cyanobacteria	NS	F	Wild feeding on cyanobacteria	[184]
Capnioneura mitis Despax, 1932	A	Rivers	Nat	Cyanobacteria	NS	F	Wild feeding on cyanobacteria; gut contents analysis	[185]
**Arthropoda, Hexapoda, Plecoptera, Leuctridae**								
Leuctra andalusiaca Aubert, 1962	A	River	Nat	Cyanobacteria	NS	F	Wild feeding on cyanobacteria; gut contents analysis	[185]
Leuctra digitata Kempny, 1899	L	Rivers	Nat	*Tolypothrix tenuis*, *Calothrix gypsophila*, *Coelosphaerium kuetzingianum*, *Gomphosphaeria lacustris*	NS	F	Wild feeding on cyanobacteria; Gut contents analysis	[160]

	Leuctra franzi Aubert, 1956	A	River	Nat	Cyanobacteria	NS	F	Wild feeding on cyanolichens; gut contents analysis	[185]
	Leuctra fusca (Linnaeus, 1758)	A, L	River	Nat	Cyanobacteria; *Tolypothrix tenuis, Calothrix gypsophila, Coelosphaerium kuetzingianum, Gomphosphaeria lacustris*	NS	F	Wild feeding on cyanobacteria; gut contents analysis	[160,185]
	Leuctra geniculata (Stephens, 1836)	L	River	Nat	Cyanobacteria	NS	F	Wild feeding on cyanobacteria; gut contents analysis; Effects on larval growth	[179]
	Leuctra iliberis Sánchez-Ortega & Alba-Tercedor, 1988	A	River	Nat	Cyanobacteria	NS	F	Wild feeding on cyanobacteria; gut contents analysis	[185]
	Leuctra inermis Kempny, 1899	A	River	Nat	Cyanobacteria	NS	F	Wild feeding on cyanolichens; gut contents analysis	[185]
	Leuctra maroccana Aubert, 1956	A	River	Nat	Cyanobacteria	NS	F	Wild feeding on cyanobacteria; gut content sanalysis	[185]
	Tyrrhenoleuctra spp.	L	Rivers	Nat	Cyanobacteria	NS	F	Wild feeding on cyanobacteria; gut contents analysis; Effects on larval growth	[186]
**Arthropoda, Hexapoda, Plecoptera, Chloroperlidae**									
	Chloroperla nevada Zwick, 1967	A	Rivers	Nat	Cyanobacteria	NS	F	Wild feeding on cyanobacteria; gut contents analysis	[187]
	Isoptena serricornis (Pictet, 1841)	A	Rivers	Nat	Cyanobacteria	NS	F	Wild feeding on cyanobacteria; gut contents analysis	[188]
	Sweltsa sp.	L	Streams	Lab	*Nostoc parmelioides*	NS	Co	Tolerance habitats abundant in cyanobacteria	[113]
**Arthropoda, Hexapoda, Plecoptera, Nemouridae**									
	Amphinemura triangularis (Ris, 1902)	A	Rivers	Nat	Cyanobacteria	NS	F	Wild feeding on cyanobacteria; gut contents analysis	[185]
	Protonemura alcazaba (Aubert, 1954)	A	Rivers	Nat	Cyanobacteria	NS	F	Wild feeding on cyanobacteria; gut contents analysis	[185]
	Protonemura meyeri (Pictet, 1842)	A	Rivers	Nat	Cyanobacteria	NS	F	Wild feeding on cyanobacteria; gut contents analysis	[185]
	Nemoura cinerea (Retzius, 1783)	A	Rivers	Nat	Cyanobacteria	NS	F	Wild feeding on cyanobacteria; gut contents analysis	[185]
	Nemoura fulviceps Klapálek, 1902	A	Rivers	Nat	Cyanobacteria	NS	F	Wild feeding on cyanobacteria; gut contents analysis	[185]
	Visoka cataractae (Neave, 1933)	larvae	Rivers	Nat	*Nostoc parmelioides*	NS	Co	Tolerance habitats abundant in cyanobacteria	[113]
	Zapada sp	larvae	Rivers	Nat	*Nostoc parmelioides*	NS	Co	Tolerance habitats abundant in cyanobacteria	[113]
**Arthropoda, Hexapoda, Plecoptera, Peltoperlidae**									
	Yoraperla sp	L	Streams	Lab	*Nostoc parmelioides*	NS	Co	Tolerance habitats abundant in cyanobacteria	[113]
**Arthropoda, Hexapoda, Plecoptera, Perlodidae**									

Hemimelaena flaviventris (Pictet, 1842)	A	Rivers	Nat	Cyanobacteria	NS	F	Wild feeding on cyanobacteria; gut contents analysis	[189]
Isoperla nevada Banks, 1906	A	Rivers	Nat	Cyanobacteria	NS	F	Wild feeding on cyanobacteria; gut contents analysis	[187]
Megarcys sp.	L	Streams	Lab	*Nostoc parmelioides*	NS	Co	Tolerance habitats abundant in cyanobacteria	[113]
**Arthropoda, Hexapoda, Plecoptera, Taeniopterygidae**								
Taeniopteryx nivalis (Fitch, 1847)	A	Rivers	Nat	Cyanobacteria	NS	F	Wild feeding on cyanobacteria	[183,184]
Taeniopteryx parvula Banks, 1918	A	Rivers	Nat	Cyanobacteria	NS	F	Wild feeding on cyanobacteria	[184]
Strophopteryx fasciata (Burmeister, 1839)	A	Rivers	Nat	Cyanobacteria	NS	F	Wild feeding on cyanobacteria	[184]
**Arthropoda, Hexapoda, Odonata, Calopterygidae**								
Calopteryx Leach, 1815	L	Lakes	Lab	*Microcystis aeruginosa (+)*	NS	CB	Survival/mortality	[153]
**Arthropoda, Hexapoda, Odonata, Coenagrionidae**								
Enallagma Charpentier, 1840	L	Lakes	Lab	*Microcystis aeruginosa (+)*	NS	CB	Survival/mortality	[153]
**Arthropoda, Hexapoda, Odonata, Lestidae**								
Lestes Leach, 1815	L	Lakes	Lab	*Microcystis aeruginosa (+)*	NS	CB	Survival/mortality	[153]
**Arthropoda, Hexapoda, Trichoptera, Hydropsychidae**								
Arctopsyche grandis (Banks, 1900)	L	Streams	Lab	*Nostoc parmelioides*	NS	Co	Tolerance habitats abundant in cyanobacteria	[113]
Hydropsyche angustipennis (Curtis, 1834)	L	Rivers	Nat	*Tolypothrix tenuis, Calothrix gypsophila, Coelosphaerium kuetzingianum, Gomphosphaeria lacustris*	NS	F	Wild feeding on cyanobacteria; Gut contents analysis	[160]
Hydropsyche instabilis (Curtis, 1834)	L	Rivers	Nat	*Tolypothrix tenuis, Calothrix gypsophila, Coelosphaerium kuetzingianum, Gomphosphaeria lacustris*	NS	F	Wild feeding on cyanobacteria; Gut contents analysis	[160]
**Arthropoda, Hexapoda, Trichoptera, Rhyacophila**								
Rhyacophila acropedes Banks, 1914	L	Streams	Lab	*Nostoc parmelioides*	NS	Co	Tolerance habitats abundant in cyanobacteria	[113]
Rhyacophila tucula Ross, 1950	L	Streams	Lab	*Nostoc parmelioides*	NS	Co	Tolerance habitats abundant in cyanobacteria	[113]
Rhyacophila vaccua Milne, 1936	L	Streams	Lab	*Nostoc parmelioides*	NS	Co	Tolerance habitats abundant in cyanobacteria	[113]
**Arthropoda, Hexapoda, Trichoptera, Psychomyidae**								
Psychomyia pusilla (Fabricius, 1781)	L	Streams	Nat	Cyanobacteria oncoids	NS	Co	Inhabit colonies, mats or mass of cyanobacteria	[119]
**Arthropoda, Hexapoda, Trichoptera, Helicopsychidae**								

Helicopsyche sp.	L	Streams	Nat	*Dolichospermum sp.*	NS	F	Wild feeding on cyanobacteria; Effect on algal community	[190]
**Arthropoda, Hexapoda, Trichoptera, Hydroptilidae**								
Leucotrichia pictipes (Banks, 1911)	L	Streams	Nat	*Periphyton: Schizothrix calcicola, Microcoleus vaginatus*	NS	F, Co	Wild feeding on cyanobacteria; Gut contents analysis; Food selection analysis; Inhabit colonies, mats or mass of cyanobacteria	[77]
Oxyethira albiceps (McLachlan, 1862)	L	Rivers	Nat	Patches of periphyton: *Phormidium* sp./*Oscillatoria* sp.	NS	Co	Inhabit colonies, mats or mass of cyanobacteria	[191]
**Arthropoda, Hexapoda, Hemiptera, Corixidae**								
Cenocorixa bifida (Hungerford, 1926)	L, A	Not indicated	Lab+Nat	Cyanobacteria	NS	F	Wild feeding on cyanobacteria; Gut contents analysis	[83,192]
Cenocorixa expleta (Uhler, 1895)	L, A	Not indicated	Lab+Nat	Cyanobacteria	NS	F	Wild feeding on cyanobacteria; Gut contents analysis	[83,192]
Corixa Leach, 1815	L, A	Not indicated	Lab+Nat	*Oscillatoria sp.*	NS	F	Wild feeding on cyanobacteria; Gut contents analysis	[83,193]
Ramphocorixa acuminiata (Uhler, 1897)	L, A	Not indicated	Lab+Nat	*Oscillatoria* sp., *Anabaena* sp., *Merismopedia* sp.	NS	F	Wild feeding on cyanobacteria; Gut contents analysis	[83,194]
Sigara striata Linnaeus, 1758	L, A	Not indicated	Lab+Nat	*Anabaena* sp. and allied algae	NS	F	Wild feeding on cyanobacteria; Gut contents analysis	[83,195]
**Arthropoda, Hexapoda, Coleoptera, Elmidae**								
Elmidae	L	Rivers	Nat	Patches of periphyton: *Phormidium* sp./*Oscillatoria* sp.	NS	Co	Inhabit colonies, mats or mass of cyanobacteria	[191]
**Arthropoda, Crustacea, Amphipoda**								
Echinogammarus ischnus (Stebbing, 1899)	U	Lakes	Nat	*Microystis aeruginosa* (+); MCs from sediments	MCs	F, CF	Wild feeding on cyanobacteria; Toxins bioaccumulation; Food web toxins transfer	[107,134]
Gmelinoides fasciatus (Stebbing, 1899)	7-12 mm length	Reservoir	Lab+Nat	*Aphanizomenon flos-aquae (90%), Microcystis aeruginosa* (10%)	MC-LR, -YR, -RR; ANTX-a,	F, CF	Wild feeding on cyanobacteria; Food assimilation analysis (isotope labeling 13C and 15N); Survival/mortality; Harmful effect on immature (embryos) and physiology (heart tolerance); Toxicity synergies	[81,178]
Hyalella azteca (Saussure, 1858)	U	Lakes	Lab+Nat	*Phormidium* sp (strain 1 (+): *P.* cf. *subfuscum*; strain 2 (+): *P.* cf. *irriguum;* strain 3 (+): *P.* cf. *autumnale*); Cyanoperiphyton; *Lyngbya* sp.	ANTX; NS	F, CF	Forced feeding on cyanobacteria; Survival/mortality; Food assimilation analysis (isotope labeling); Survival/mortality; Harmful effect of extracts; effective refuge against predator; Inhabit colonies, mats or mass of cyanobacteria	[95,154,196]
**Arthropoda, Crustacea, Isopoda**								
Asellus aquaticus (Linnaeus, 1758)	5-7 mm	Reservoir	Lab+Nat	*Aphanizomenon* sp.	NS	F	Wild feeding on cyanobacteria; Food assimilation analysis (isotope labeling 13C and 15N);	[178]
**Arthropoda, Crustacea, Decapoda, Atyidae**								

Caridina denticulata (De Haan, 1844)	U	Artificial pond		*Microcystis aeruginosa*, *Anabaena spiroides*, *Synechocystis pevalekii*, *Aphanocapsa elachista*, *Chroococcus sp.*	MC-LR, -RR, -YR	F	Food assimilation analysis (isotope labeling 13C and 15C); Bioremediation (cyanobacteria control and detoxification in nature)	[197]
**Arthropoda, Crustacea, Decapoda, Palaemonidae**								
Macrobrachium nipponensis (De Haan, 1849)	66.5±3.5 mm length	Lakes	Nat	Cyanobacteria blooms	MC-LR, MC-RR	F	Wild feeding on cyanobacteria; Toxin Bioaccumulation and target organs; Foodweb toxin transfer; Toxin seafood problem on public health	[99]
Macrobrachium rosenbergii (De Man, 1879)	8,36-8,65 cm length and 12,92-13,87 g weight; 5-month-old	Breeding farm	Lab	*Microcystis aeruginosa* (+)	MC-LR		Forced feeding on cyanobacteria; Toxins bioaccumulation and target organs; Food web toxin transfer; survival/mortality; Harmfull effect on cell, tissues and molecular metabolism	[100,121]
Palaemon modestus (Heller, 1862)	62±4.5 mm length	Lakes	Nat	Cyanobacteria blooms	MC-LR, -RR	F	Wild feeding on cyanobacteria; Toxin Bioaccumulation; Target organs; Food web toxin transfer	[99]
Palaemonetes argentinus Nobili, 1901	31.6±6.2 mm length, 0.15±0.03 g weight	Reservoir	Lab+Nat	Cyanobacteria blooms	MC-LR, -RR, -LA, -YR; NOD	CF	Toxin bioaccumulation; Metabolic detoxification processes (stress oxidative enzymes: GST, GR, GPX and CAT)	[128]
**Arthropoda, Crustacea, Decapoda, Astacidae**								
Astacus astacus (Limnaeus, 1758)	U	Lale		*Planktothrix agardhii*, *P. rubescens*	MC-RR, -LR, -YR, -LA, -LY, -LF, -LW	U	Toxins bioaccumulation; Target organs; Toxins seafood problems on public health	[198]
Cherax quadricarinatus (Von Martens, 1868)	A	Breeding farm	Lab+Nat	*Cylindrospermopsis raciborskii*	CYN	F, CF	Wild feeding on cyanobacteria; Gut content analysis; Toxins bioaccumulation; target organs; Long-term toxin exposition analysis.	[122]
Pacifastacus leniusculus (Dana, 1852)	A (44,2 mm length and 30-19 g weight)	Pond and Breeding farm	Lab+Nat	*Oscillatoria sancta (+) and Planktothris agardhii (+ and -)*	MCs	F	Wild feeding on cyanobacteria; Gut contents analysis; Survival/mortality; Toxins bioaccumulation; Harmful effect on behavior and growth	[199]
Paranephrops planifrons White, 1842	J	Lakes	Lab	Cyanobacteria mats (+); *Microcystis* sp. (extracts and cells)	MC-AR, -FR, -LA, -LR (dm-LR), -RR (dm-RR), -WR, -YR; NOD	F, CF	Forced feeding on cyanobacteria; Survival/mortality; Food assimilation analysis (isotope labeling); Toxins bioaccumulation; Target organs; Harmful effects on behavior; Toxins seafood problems on public health	[30,200]
Paranephrops zealandicus (White, 1847)	U	Streams	Nat	*Nostoc* sp.	NS	F	Wild feeding on cyanobacteria; Gut contents and food assimilation (isotope labeling) analysis	[87]
Procambarus clarkii (Girard, 1852)	L, J, A (size very variable among papers)	Lakes; ponds; breeding farm	Lab+Nat	*Microcystis aeruginosa* (+ and -); *Anabaena spiroides*; Not identified	Extracts; MC-LR, -RR, -YR	F, CF	Forced feeding on cyanobacteria; Survival/mortality; Toxins bioaccumulation; Target organs; Food antioxidants and toxins blockers (astaxanthin); Harmful effect of not toxic strains cyanobacteria; Harmful effect	[32,99,123,133,201]

							on cell, tissue, and gut microbiota; Depuration; Metabolic detoxification processes (transcriptomic identification of immune and redox related DEGs); Food web toxin transfer; Toxins seafood problems on public health; New methods for detecting cyanotoxins	
**Arthropoda, Crustacea, Ostracoda, Astacidae; Cyprididae**								
Cyprinotus incongruens (Ramdohr, 1808)	L, J, A	not indicated	Lab	*Aphanizomenon flos-aquae* (-), *Anabaena affinis* (+), *A. ambigua* (-); *A. baltica* (+), *A. catenula* (-); *A. circinalis* (+), *A. doliolum* (+), *A. flos-aquae* (-), *A. inaequalis* (+), *A. laza*, *A. levanderi* (+), *A. limnetica* (-), *A. macrospora* (-), *A. orientalis* (-), *A. randhawae*, *A. sphaerica* (-), *A. torulosa* (+, -); *A. variabilis* (+), *Chroococcus* sp., *Cylindrospermum doryphorum* (+), *Fischerella epiphytica* (+), *F. muscicola* (+), *Fremyella* sp. (+), *Gloeotrichia echinutata* (+), *Gloeotrichia* sp., *Microcystis aeruginosa* (+, -), *Nostoc coeruleum* (+), *N. cuticulare* (+), *N. pruniforme* (-), *N. macrosporum* (-), *N. rivulare* (+), *N. spongiaeforme* (-), *N. zetterstedtii* (+), *Nostoc* sp. (-), *Oscillatoria agardhii* (+), *O. lacustris* (+), *Tolypothrix distorta* (+), *Westiella intricata* (-)	NS	F, CB	Forced feeding on cyanobacteria; Survival/mortality; Harmful effect on life cycle and behavior; Food selection analysis	[130]
Dolerocypris fasciata (O.F. Müller, 1776)	U	Reservoir	Nat	Cyanobacteria	NS	F	Wild feeding on cyanobacteria; Life cycle	[83,202]
**Mollusca, Bivalvia, Cyrenidae**								
Corbicula fluminea (O.F. Müller, 1774)	2.1 ± 0.1 cm width, 2.01 ± 0.1 cm height	Lakes	Nat	*Microcystis* sp., *Anabaena* sp.; *Microcystis aeruginosa* (+); *Pseudanabaena mucicola*; *Nostoc* sp., *Anabaena cylindrica*, *Desmonostoc muscorum*, *Pseudanabaena* sp.	MC-RR, -LR	F	Toxin bioaccumulation; Target organs; Toxins seafood problems on public health; Toxins digestion and bioaccessibility; Physical treatments to eliminate toxins in seafood; Bioremediation (cyanobacteria control and detoxification in nature); Selective filtration of cyanobacteria; Physiological detoxification processes (pseudofaeces)	[124,203,204]
Corbicula javanica (Mousson, 1849)	23– 25 mm	Captive breeding	Lab	Not indicated	BMMA	CF	Toxin bioaccumulation; Forced uptake of cyanotoxins; Physiological detoxification processes (faeces); Depuration	[205]

Corbicula leana Prime, 1864	2.46 ± 0.57 cm length	Captive breeding		Cyanobacteria crude extracts	MC-RR, -LR, -YR	CF	Toxin bioaccumulation; target organs; Metabolic detoxification processes (stress oxidative enzymes: CAT, SOD, GSTs); Depuration	[206]
Corbicula sandai Reinhardt, 1878	U	Lakes	Lab+Nat	*Microcystis aeruginosa, Oscillatoria kawamurae, Anabaena spiroides*	MC-LR, -RR	CF, CB	Toxin bioaccumulation; Target organs; Depuration; Field/laboratory data comparison; Food web toxin transfer	[207]
**Mollusca, Bivalvia, Dreissenidae**								
Dreissenid	U	Lakes	Nat	*Microcystis* sp.	MCs	Co	Territorial and seasonal distribution conditioned by cyanobacteria	[208]
Dreissena polymorpha (Pallas, 1771)	A (15-30 mm); veliger	Lakes; Rivers; Reservoir	Lab+Nat	*Anabaena flos-aquae* (+), *Aphanizomenon flos-aquae* (-), *Dolichospermum lemmermanii* (-), *Gloeotrichia echinulata* (-), *Microcystis aeruginosa* (+, -), *M. wesenbergii* (-), *Planktothrix suspensa* (+); Not indicated	MCs; MC-LR, -RR, -YR, -LF; BMMA; NOD	F, CF, CB	Wild and Forced feeding/uptake on cyanobacteria; Toxins in sediments analysis; Toxins bioaccumulation; Food web toxins transfer; Metabolic detoxification processes (stress oxidative enzymes: GST, CAT, SOD); Physiological detoxification processes (faeces or pseudofaeces/pseudodiarrhoea); Depuration; Harmful effects on growth, feeding behavior, cell, tissue, molecular (DNA damage) and immune functions	[25,28,132,205,209, 210,211,212,213,214,215,216,217,218, 219]
Dreissena rostriformis bugensis Andrusov, 1897	Veliger	Rivers	Lab	*Anabaena flos-aquae* (+), *Aphanizomenon flos-aquae* (-), *Dolichospermum lemmermanii* (-), *Gloeotrichia echinulata* (-), *Microcystis aeruginosa* (+, -), *M. wesenbergii* (-), *Planktothrix suspensa* (+)	MCs	CF, CB	Forced feeding on cyanobacteria; Survival/mortality; Harmful effect of not-toxic strains;	[210]
**Mollusca, Bivalvia, Mytilidae**								
Limnoperna fortunei (Dunker, 1857)	Veliger	Reservoir	Lab+Nat	cyanobacteria bloom: *Microcystis* spp.	MC-LR	Co	Abundance conditioned by cyanobacteria; Survival/mortality	[220]
**Mollusca, Bivalvia, Sphaeriidae**								
Psidium sp.	U	Lakes	Nat	*Anabaena heterospora* (+), *A. circinalis* (+), *Aphanizomenon flos aquae* (+), *A. issatchenkoi* (+), *Aphanothece* sp., *Coelomoron* sp., *Limnotrix redekei*, *Microcystis aeruginosa* (+), *M. flos aquae* (+), *M. wesenbergii* (+), *Microcystis* sp. (+), *Planktothrix agardhii* (+), *Trichodesmium* sp. (+), *Woronichinia* sp.	NS	F, CF, CB	Toxin bioaccumulation; Harmful effect on community structure	[221]
Pisidium amnicum (O. F. Müller, 1774)	U	Lakes	Nat	*Anabaena heterospora* (+), *A. circinalis* (+), *Aphanizomenon flos aquae* (+), *A. issatchenkoi* (+), *Aphanothece* sp., *Coelomoron* sp.,	NS	F, CF, CB	Toxin bioaccumulation; Harmful effect on community structure	[221]

				Limnotrix redekei, *Microcystis aeruginosa* (+), *M. flos aquae* (+), *M. wesenbergii* (+), *Microcystis* sp. (+), *Planktothrix agardhii* (+), *Trichodesmium* sp. (+), *Woronichinia* sp.				
Pisidium casertanum (Poli, 1791)	U	Rivers	Nat	*Tolypothrix tenuis*, *Calothrix gypsophila*, *Coelosphaerium kuetzingianum*, *Gomphosphaeria lacustris*	NS	F	Wild feeding on cyanobacteria; Gut contents analysis	[160]
Sphaerium corneum (Linnaeus, 1758)	U	Lakes	Nat	*Anabaena heterospora* (+), *A. circinalis* (+), *Aphanizomenon flos aquae* (+), *A. issatchenkoi* (+), *Aphanothece* sp., *Coelomoron* sp., *Limnotrix redekei*, *Microcystis aeruginosa* (+), *M. flos aquae* (+), *M. wesenbergii* (+), *Microcystis* sp. (+), *Planktothrix agardhii* (+), *Trichodesmium* sp. (+), *Woronichinia* sp.	NS	F, CF, CB	Toxin bioaccumulation; Harmful effect on community structure	[221]
**Mollusca, Bivalvia, Unionidae**								
Anodonta anatina (Linnaeus, 1758)	U	Rivers	Nat	Not indicated	MC-LR, -RR, -YR, -LA, LY, -LW, -LF, dmMC-LR, dmMC-RR	CF	Toxin bioaccumulation (free and protein-bound); Food web toxin transfer; Toxin tracking along river system	[125]
Anodonta cygnea (Linnaeus, 1758)	70–90 mm	Captive breeding; Lakes	Lab	Not indicated; *Cylindrospermopsis raciborskii*; *Aphanizomenon issatschenkoi*; *Oscillatoria agardhii*	BMMA; CYN; PST	CF, CB	Toxin bioaccumulation; Long-term bioaccumulation; Target organs; Forced feeding/uptake of cyanobacteria/toxins; Physiological detoxification processes (faeces); Depuration; Harmful effects on feeding behavior; Survival/mortality	[205,222,223,224,225]
Anodonta grandis Say, 1829	65–75 mm length	Lakes	Lab+Nat	Not indicated	MC-LR	CF	Toxin bioaccumulation; Target organs; Depuration	[226]
Anodonta woodiana (I. Lea, 1834)	130 ± 8.4 mm length	Lakes	Lab	*Microcystis* sp.	MC-LR, -YR, -RR	F, CF	Toxin bioaccumulation and seasonal variation; Target organs; Field/laboratory data comparison; Food assimilation analysis (isotope labeling); Toxins seafood problems on public health; Metabolic detoxification processes (oxidative stress enzymes: GST)	[227,228,229]
Arconaia lanceolata (I. Lea, 1856)	U	Lakes	Nat	*Microcystis* sp., *Anabaena* sp.	MCs	CF	Toxin bioaccumulation; Target organs	[124]
Cristaria plicata (Leach, 1814)	240 ± 13,5 mm length	Lakes	Lab	*Microcystis* sp.	MC-LR, -YR, -RR	CF	Toxin bioaccumulation and seasonal variation; Target organs; Field/laboratory data comparison; Food	[227,228,229,230,231]

								assimilation analysis (isotope labeling); Toxins seafood problems on public health; Metabolic detoxification processes: oxidative stress enzymes (GST) and activation of signaling pathways protects against oxidative damage (Nrf2/Keap1);	
	Echyridella menziesii (J. E. Gray, 1843)	J (11 ± 2 mm, 0.13 ± 0.07 g)	Lakes	Lab	*Microcystis* sp. (extracts)	MC-RR, -YR, -LR, -FR, -WR, -AR, -LA, dmMC-RR; dmMC-LR	CF	Survival/Mortality; Toxins bioaccumulation; Target organs; Harmful effects on behavior	[30]
	Elliptio complanata (Lightfoot, 1786)	U	Lakes; Ponds	Nat	Cyanobacteria bloom	MCs	F, CF	Wild feeding cyanobacteria; Toxins bioaccumulation; Target organs; Depuration	[232]
	Hyriopsis cumingii (I. Lea, 1852)	190 ± 12,4 mm length	Lakes	Lab	*Microcystis* sp.; *Microcystis aeruginosa*	MC-LR, -YR, -RR	CF	Toxin bioaccumulation; Target organs; Field/laboratory data comparison; Food assimilation analysis (isotope labeling and fatty acids marking); Toxins seafood problems on public health; Metabolic detoxification processes (Antioxidant response: SOD, CAT, GPx, GST, ROS, MDA and GSH); Toxicity synergies	[129,233]
	Lamprotula leaii (J. E. Gray, 1833)	100 ± 6.5 mm length	Lakes	Lab	*Microcystis* sp.	MC-LR, -YR, -RR	CF	Toxins bioaccumulation; Target organs; Field/laboratory data comparison; Food assimilation analysis (isotope labeling); Toxins seafood problems on public health; Metabolic detoxification processes (oxidative stress enzymes: GST)	[233]
	Lampsilis radiata (Gmelin, 1791)	U	Lakes; Ponds	Nat	Cyanobacteria bloom	MCs	F, CF	Wild feeding cyanobacteria; Toxins bioaccumulation; Target organs; Depuration	[232]
	Leptodea ochracea (Say, 1817)	U	Lakes; Ponds	Nat	Cyanobacteria bloom	MCs	F, CF	Wild feeding cyanobacteria; Toxins bioaccumulation; Target organs; Depuration	[232]
	Pyganodon cataracta (Say, 1817)	U	Lakes; Ponds	Nat	Cyanobacteria bloom	MCs	F, CF	Wild feeding cyanobacteria; Toxins bioaccumulation; Target organs; Depuration	[232]
	Sinanodonta arcaeformis	U	Artificial pond		*Microcystis aeruginosa*, *Anabaena spiroides*, *Synechocystis pevalekii*, *Aphanocapsa elachista*, *Chroococcus sp.*	MC-LR, -RR, -YR	F	Food assimilation analysis (isotope labeling 13C and 15C); Bioremediation (cyanobacteria control and detoxification in nature)	[232]
	Unio douglasiae J. E. Gray, 1833	U	Artificial pond		*Microcystis aeruginosa*, *Microcystis* sp., *Anabena spiroides*, *Synechocystis pevalekii*, *Aphanocapsa elachista*, *Chroococcus sp.*	MC-LR, -RR, -YR	F	Toxin bioaccumulation and seasonal variation; Target organs; Food assimilation analysis (isotope labeling 13C and 15C); Bioremediation (cyanobacteria control and detoxification in nature); Depuration; Toxins seafood problems on public health	[197,228,229,234]
	Unio pictorum (Linnaeus, 1758)	70–89 mm length	Reservoir	Lab+Nat	*Aphanizomenon flos-aquae (90%)*, *Microcystis aeruginosa* (10%)	MC-LR, -YR, -RR; ANTX-a,	CF	survival/mortality; Harmful effect on immature (embryos) and physiology (heart tolerance, heart rate and its recovery); Toxicity synergies	[178]

Unio tumidus Philipsson, 1788	50–90 mm length	Lakes	Lab	*Microcystis aeruginosa* (extracts); Not indicated	MC-LR, extracts; BMMA	F, CF	Wild feeding in cyanobacteria; Toxins bioaccumulation; Metabolic detoxification processes (stress oxidative enzymes: GST, CAT, SOD); Physiological detoxification processes (faeces); Depuration; Food assimilation analysis (fatty acids biomarkers)	[205,211,215]
**Mollusca, Gastropoda, Heterobanchia, Physidae**								
Aplexa hypnorum (Linnaeus, 1758)	U	Lakes	Nat	*Anabaena heterospora* (+), *A. circinalis* (+), *Aphanizomenon flos aquae* (+), *A. issatchenkoi* (+), *Aphanothece* sp., *Coelomoron* sp., *Limnotrix redekei*, *Microcystis aeruginosa* (+), *M. flos aquae* (+), *M. wesenbergii* (+), *Microcystis* sp. (+), *Planktothrix agardhii* (+), *Trichodesmium* sp. (+), *Woronichinia* sp.	NS	Co	Toxin bioaccumulation; Harmful effect on community structure	[221]
Physa acuta Draparnaud, 1805	J, A	Reservoir; Lakes	Nat	*Anabaena* sp., *Aphanizomenon* sp., *Microcystis* sp., *Planktothrix* sp., *Trichodesmium* sp.; Patches of periphyton: *Phormidium* sp./*Oscillatoria* sp; *Anabaena flos-aquae* (+), *Anabaena planktonica*, *A. spiroides* (+), *Aphanizomenon flos-aquae*, *A. gracile*, *Aphanocapsa* sp., *Aphanothece* sp., *Coelomoron* sp., *Coelosphaerium* sp., *Limnothrix redekeii*, *Merismopedia* sp., *Microcystis aeruginosa* (+), *M. flos-aquae* (+), *Microcystis wesenbergii* (+), *Oscillatoria* sp. (+), *Plankthotrix agardhii* (+), *Pseudanabaena catenata*, *P. limnetica*, *Spirulina* sp. *Synechoccochus* sp., *Woronichia* sp.	MCs	F, CF, Co	Seasonal abundance and maturation; Toxins bioaccumulation related with season; Target organs; Toxicity synergies; Inhabit mats (periphyton) of cyanobacteria; Territorial and seasonal distribution conditioned by cyanobacteria	[78,191,235]
Physa gyrina Say, 1821	U	Lakes	Nat	*Microcystis aeruginosa*	MC-LR	CF, CB	Toxin bioaccumulation	[236,237]
**Mollusca, Gastropoda, Heterobanchia, Planorbidae**								
Ancylus fluviatilis (O.F. Müller, 1774)	J, A	Reservoir	Nat	Most frecuent genera in study area: *Anabaena* sp., *Aphanizomenon* sp., *Microcystis* sp., *Planktothrix* sp., *Trichodesmium* sp.	MCs	F, CF	Toxin bioaccumulation; Harmful effect on community structure	[78,221]
Armiger crista (Linnaeus, 1758)	U	Lakes	Nat	*Anabaena heterospora* (+), *A. circinalis* (+), *Aphanizomenon flos aquae* (+), *Microcystis flos aquae* (+),	NS	F, CF, CB	Toxin bioaccumulation; Harmful effect on community structure	[221]

				M. viridis (+), *Oscillatoria* sp. (+), *Planktothrix agardhii* (+)				
	Gyraulus albus (O. F. Müller, 1774)	U	Lakes	Nat	*Anabaena heterospora* (+), *A. circinalis* (+), *Aphanizomenon flos aquae* (+), *A. issatchenkoi* (+), *Aphanothece* sp., *Coelomoron* sp., *Limnotrix redekei*, *Microcystis aeruginosa* (+), *M. flos aquae* (+), *M. wesenbergii* (+), *Microcystis* sp. (+), *Planktothrix agardhii* (+), *Trichodesmium* sp. (+), *Woronichinia* sp.	NS	F, CF, CB	Toxin bioaccumulation; Harmful effect on community structure	[221]
	Helisoma trivolvis (Say, 1817)	U	Lakes	Nat	*Microcystin aeruginosa*	MC-LR	CF, CB	Toxin bioaccumulation	[236,237]
	Hippeutis complanatus (Linnaeus, 1758)	J, A	Reservoir	Nat	Most frecuent genera in study area: *Anabaena* sp., *Aphanizomenon* sp., *Microcystis* sp., *Planktothrix* sp., *Trichodesmium* sp.	MCs	F, CF	Toxin bioaccumulation; Harmful effect on community structure	[78,221]
	Planorbis planorbis (Linnaeus, 1758)	J, A	Reservoir	Nat	Most frecuent genera in study area: *Anabaena* sp., *Aphanizomenon* sp., *Microcystis* sp., *Planktothrix* sp., *Trichodesmium* sp.; *Anabaena flos-aquae* (+), *A. planktonica*, *A. spiroides* (+), *A. heterospora* (+), *A. circinalis* (+), *Aphanizomenon flos-aquae, A. gracile*, *A. issatchenkoi* (+), *Aphanocapsa* sp., *Aphanothece* sp., *Coelomoron* sp., *Coelosphaerium* sp., *Limnothrix redekeii*, *Merismopedia* sp., *Microcystis aeruginosa* (+), *M. flos-aquae* (+), *M. wesenbergii* (+), *Oscillatoria* sp. (+), *Plankthotrix agardhii* (+), *Pseudanabaena catenata*, *P. limnetica*, *Spirulina* sp. *Synechoccochus* sp., *Trichodesmium* sp. (+), *Woronichinia* sp.	MCs	F, CF	Toxin bioaccumulation; Territorial and seasonal distribution conditioned by cyanobacteria; Harmful effect on community structure	[78,221,235]
**Mollusca, Gastropoda, Heterobanchia, Lymnaeidae**								
	Lymnaea stagnalis (Linnaeus, 1758)	J (14 ± 1 mm length), A (25-50 mm height)	Captive breeding; lakes	Lab	*Planktothrix agardhii (+, -); Anabaena flos-aquae (+), A. planktonica, A. spiroides (+), Aphanizomenon flos-aquae, A. gracile, Aphanocapsa sp., Aphanothece sp., Coelomoron sp.,*	MC-RR, dmMC-RR, dmMc-LR, MC-YR	F, CF, CB	Forced feeding on cyanobacteria; Food selection analysis; Toxin bioaccumulation (free and covalently bound); Target organs; Depuration; Physiological detoxification processes (egestion); Metabolic detoxification processes (stress oxidative enzymes: GST and CAT); Harmful effects on life cycle, embryo and	[235,236,237,238,239,240,241,242,243,244,245,246]

					Coelosphaerium sp., Limnothrix redekeii, Merismopedia sp., Microcystis aeruginosa (+), M. flos-aquae (+), M. wesenbergii (+), Oscillatoria sp. (+), Plankthotrix agardhii (+), Pseudanabaena catenata, P. limnetica, Spirulina sp. Synechococcus sp., Woronichia sp.			immature growth, fecundity, hatching, cell, tissue, population, and behavior; Survival/mortality; Food web Toxin transfer; Differential toxins uptake analysis; Field/laboratory data comparison	
	Radix auricularia (Linnaeus, 1758)	J, A	Reservoir	Nat	Most frecuent genera in study area: *Anabaena* sp., *Aphanizomenon* sp., *Microcystis* sp., *Planktothrix* sp., *Trichodesmium* sp.; *Anabaena heterospora* (+), *Anabaena circinalis* (+), *Aphanizomenon flos aquae* (+), *A. issatchenkoi* (+), *Aphanothece* sp., *Coelomoron* sp., *Limnotrix redekei*, *Microcystis aeruginosa* (+), *M. flos aquae* (+), *M. wesenbergii* (+), *M. viridis* (+), *Microcystis* sp. (+), *Oscillatoria* sp. (+); *Planktothrix agardhii* (+), *Trichodesmium* sp. (+), *Woronichinia* sp.	MCs	F, CF	Toxin bioaccumulation; Harmful effect on community structure	[78,221]
	Radix ovata (Draparnaud, 1805)	J, A	Reservoir; Lakes	Nat	Most frecuent genera in study area: *Anabaena* sp., *Aphanizomenon* sp., *Microcystis* sp., *Planktothrix* sp., *Trichodesmium* sp.; *Anabaena flos-aquae* (+), *A. planktonica*, *Anabaena heterospora* (+), *A. circinalis* (+), *A. spiroides* (+), *Aphanizomenon flos-aquae*, *A. gracile*, *Aphanocapsa* sp., *Aphanothece* sp., *Coelomoron* sp., *Coelosphaerium* sp., *Limnothrix redekeii*, *Merismopedia* sp., *Microcystis aeruginosa* (+), *M. flos-aquae* (+), *Microcystis wesenbergii* (+), *Oscillatoria* sp. (+), *Plankthotrix agardhii* (+), *Pseudanabaena catenata*, *P. limnetica, Spirulina* sp. *Synechococcus* sp., *Trichodesmium* sp., *Woronichia* sp.	MCs	F, CF	Toxin bioaccumulation; Territorial and seasonal distribution conditioned by cyanobacteria; Harmful effect on community structure	[78,221,235]
	Radix swinhoei (H. Adams, 1866)	36.0 x 24.0 mm in size	Lakes	Lab+Nat	Not identified	MCs	CF, CB	Toxin bioaccumulation; Target organs; Field/laboratory data comparison	[247]
**Mollusca, Gastropoda, Heterobranchia, Valvatidae**									

Valvata cristata O. F. Müller, 1774	U	Lakes	Nat	*Anabaena flos-aquae* (+), *A. planktonica*, *A. spiroides* (+), *Aphanizomenon flos-aquae*, *A. gracile*, *Aphanocapsa* sp., *Aphanothece* sp., *Coelomoron* sp., *Coelosphaerium* sp., *Limnothrix redekeii*, *Merismopedia* sp., *Microcystis aeruginosa* (+), *M. flos-aquae* (+), *M. wesenbergii* (+), *Oscillatoria* sp. (+), *Plankthotrix agardhii* (+), *Pseudanabaena catenata*, *P. limnetica*, *Spirulina* sp. *Synechococcus* sp., *Woronichia* sp.	MCs	U	Toxin bioaccumulation: Territorial and seasonal distribution conditioned by cyanobacteria	[235]
**Mollusca, Gastropoda, Caenogastropoda, Bythiniidae**								
Bithynia tentaculata (Linnaeus, 1758)	J, A	Reservoir	Nat	Most frecuent genera in study area: *Anabaena* sp., *Aphanizomenon* sp., *Microcystis* sp., *Planktothrix* sp., *Trichodesmium* sp.; *Anabaena heterospora* (+), *Anabaena circinalis* (+), *Aphanizomenon flos aquae* (+), *Aphanothece* sp., *Coelomoron* sp., *Limnotrix redekei*, *Microcystis aeruginosa* (+), *M. flos aquae* (+), *M. wesenbergii* (+), *Microcystis* sp. (+), *Planktothrix agardhii* (+), *Woronichinia* sp.	MCs	F, CF	Toxin bioaccumulation; Harmful effects on community structure	[78,221]
**Mollusca, Gastropoda, Caenogastropoda, Baicaliidae**								
Maackia herderiana (Lindholm, 1909)	U	Lakes	Lab+Nat	*Lyngbya limnetica*	NS	F	Wild and forced feeding on cyanobacteria; Gut contents analysis; Food assimilation analysis (isotope labeling)	[248]
**Mollusca, Gastropoda, Caenogastropoda, Tateidae**								
Potamopyrgus antipodarum (J. E. Gray, 1843)	J (2 ± 0.2 mm length), A (4 ±0.2 mm length)	River	Lab+Nat	Patches of periphyton: *Phormidium* sp./*Oscillatoria* sp.; *Planktothrix agardhii (+); Anabaena flos-aquae* (+), *A. planktonica*, *A. heterospora* (+), *A. circinalis* (+), *A. spiroides* (+), *Aphanizomenon flos-aquae*, *A. gracile*, *Aphanocapsa* sp., *Aphanothece* sp., *Coelomoron* sp., *Coelosphaerium* sp., *Merismopedia* sp., *Microcystis aeruginosa* (+), *M. flos-aquae* (+), *M. wesenbergii* (+), *Oscillatoria* sp. (+), *Plankthotrix*	NS; MC-RR, -LR	F, CF, Co	Inhabit mats (periphyton) of cyanobacteria; Territorial and seasonal distribution conditioned by cyanobacteria; forced feeding on cyanobacteria; Toxins bioaccumulation; Survival/mortality; Harmful effects on life cycle, immature growth, fecundity, Community structure; Depuration; Field/laboratory data comparison	[78,221,235,249,250]

				agardhii (+), *Trichodesmium* sp. (+), *Pseudanabaena catenata*, *P. limnetica*, *Spirulina* sp., *Synechococcus* sp., *Woronichia* sp.				
**Mollusca, Gastropoda, Caenogastropoda, Thiaridae**								
Melanoides tuberculata (O. F. Müller, 1774)	U	waterways	Lab	Extracts of *Cylindrospermopsis raciborskii (+)*	CYN; deoxy-CYN	CF, CB	Toxin bioaccumulation and bioconcentration	[251]
**Mollusca, Gastropoda, Caenogastropoda, Viviparidae**								
Bellamya aeruginosa (Reeve, 1863)	13.36-16.39 mm (width), 21.64-27.58 mm (height); offsprings	Lakes	Nat	Cyanobacterial blooms (mainly composed of *Microcystis aeruginosa* and *Anabaena spiroides*)	MC-LR, -RR, -YR	F, CF, CB	Toxin bioaccumulation and seasonal and spatial dynamics; Target organs; Food web toxin transfer; Toxins seafood problems on public health; Toxicity synergies; Harmful effect on off springs	[227,252,253,254]
Margarya melanioides G. Nevill, 1877	47.0 x 39.0 mm in size	Lakes	Lab+Nat	Not identified	MCs		Toxin bioaccumulation; Target organs; Toxins seafood problems on public health; Field/laboratory data comparison	[247]
Sinotaia histrica (A. Gould, 1859)	U	Lakes	Lab+Nat	*Microcystis aeruginosa, Oscillatoria kawamurae, Anabaena spiroides; Microcystis. wesenbergii, M. ichthyoblabe and Aphanizomenon flos-aquae.*	MC-LR, -RR	CF, CB	Toxin bioaccumulation; Target organs; Depuration; Field/laboratory data comparison; Food web toxin transfer; Harmful effect on fecundity, abundance, cell and tissue; Food antioxidants and toxins blockers (naringin)	[207,255,256]
Viviparus viviparus (Linnaeus, 1758)	U	Rivers	Nat	*Tolypothrix tenuis*, *Calothrix gypsophila*, *Coelosphaerium kuetzingianum*, *Gomphosphaeria lacustris*	NS	F	Wild feeding on cyanobacteria; Gut contents analysis	[160]
**Annelida, Clitellata, Hirudinea**								
Hirudinidae	U	Lakes	Nat	Cyanobacteria bloom: *Microcystis* sp.	NS	CF, CB	Tolerance to habitats abundant in cyanobacteria; Cyanobacteria bloom; Abundance of macroinvertebrates and cyanobacteria directly proportional	[143]
**Annelida, Clitellata, Tubificida**								
Limnodilus hoffineisteri (probably *Limnodrilus hoffmeisteri* (Claparède, 1862)	U	Lakes	Nat	*Microcystis* sp., *Anabaena* sp.	MC-LR, -RR, -YR	CF	Toxin bioaccumulation	[97,124]
Stylaria lacustris (Linnaeus, 1767)	U	Rivers	Nat	*Tolypothrix tenuis*, *Calothrix gypsophila*, *Coelosphaerium kuetzingianum*, *Gomphosphaeria lacustris*	NS	F	Wild feeding on cyanobacteria; Gut contents analysis	[160]
Stylodrilus heringianus Claparede, 1862	U	Rivers	Nat	*Tolypothrix tenuis*, *Calothrix gypsophila*, *Coelosphaerium*	NS	F	Wild feeding on cyanobacteria; Gut contents analysis	[160]

				kuetzingianum, *Gomphosphaeria lacustris*				
Tubificids	U	Lakes	Nat	Cyanobacteria bloom: *Microcystis* sp.	NS	CF, CB	Tolerance to habitats abundant in cyanobacteria; Cyanobacteria bloom; Abundance of macroinvertebrates and cyanobacteria directly proportional	[143]
**Platyhelminthes, Tricladida**								
Dugesia tigrina (Girard, 1850)	U	Rivers		*Pseudanabaena limnetica*; *Microcystis aeruginosa*, *M. wesenbergii*, *Dolichospermum flos-aquae*, *Planktothrix agardhii*, *Oscillatoria angusta*	ANA, CYN, NOD, MC-RR, -LR, -LF, -LY, -LW, -YR, dmMC-RR; dmMC-LR	CF	Survival/mortality test	[257]
**Macroinvertebrate community**								
Macroinvertebrates community	L	Lakes, Rivers	Nat	Cyanobacteria: bloom (*Microcystis* sp); Algae community structure (*Nostoc* spp., *Phormidium* sp., *Microcoleus* sp.); Oncoids	NS	CF, Co	Effects on abundance, diversity, and assemblage of macroinvertebrate community; Inhabit colonies, mats, or mass of cyanobacteria; Establishment of exclusive food web	[119,145,258,259]
